# NRBP1 and TSC22D proteins affect distal convoluted tubule physiology through modulation of the WNK pathway

**DOI:** 10.1126/sciadv.adv2083

**Published:** 2025-07-16

**Authors:** Germán Magaña-Ávila, Héctor Carbajal-Contreras, Ramchandra V. Amnekar, Toby Dite, Michelle Téllez-Sutterlin, Kevin García-Ávila, Brenda Marquina-Castillo, Alejandro Lopez-Saavedra, Norma Vazquez, Eréndira Rojas-Ortega, Eric Delpire, David H. Ellison, Dario R. Alessi, Gerardo Gamba, María Castañeda-Bueno

**Affiliations:** ^1^ Department of Nephrology and Mineral Metabolism, Instituto Nacional de Ciencias Médicas y Nutrición Salvador Zubirán, Tlalpan, Mexico City, Mexico.; ^2^ Facultad de Medicina, Universidad Nacional Autónoma de México, Coyoacan, Mexico City, Mexico.; ^3^ PECEM (MD/PhD), Facultad de Medicina, Universidad Nacional Autónoma de México, Coyoacan, Mexico City, Mexico.; ^4^ MRC Protein Phosphorylation and Ubiquitylation Unit, School of Life Sciences, University of Dundee, Dow Street, Dundee DD1 5EH, UK.; ^5^ Department of Pathology, Instituto Nacional de Ciencias Médicas y Nutrición Salvador Zubirán, Tlalpan, Mexico City, Mexico.; ^6^ Unidad de Aplicaciones Avanzadas en Microscopía del Instituto Nacional de Cancerología y la Red de Apoyo a la Investigación, Universidad Nacional Autónoma de México, Tlalpan, Mexico City, Mexico.; ^7^ Tecnologico de Monterrey, Escuela de Medicina y Ciencias de la Salud, Tlalpan, Mexico City, Mexico.; ^8^ Molecular Physiology Unit, Instituto de Investigaciones Biomédicas, Universidad Nacional Autónoma de México, Coyoacan, Mexico City, Mexico.; ^9^ Department of Anesthesiology, Vanderbilt University School of Medicine, Nashville, TN, USA.; ^10^ Division of Nephrology and Hypertension, Department of Medicine, Oregon Health and Science University, Portland, OR, USA.; ^11^ VA Portland Health Care System, Portland, OR, USA.

## Abstract

The with-no-lysine (K) (WNK) kinases regulate processes such as cell volume and epithelial ion transport through the modulation of cation chloride cotransporters such as the NaCl cotransporter (NCC) present in the distal convoluted tubule (DCT) of the kidney. Recently, the interaction of WNKs with nuclear receptor binding protein 1 (NRBP1) and transforming growth factor–β–stimulated clone 22 domain (TSC22D) proteins was reported. Here, we explored the effect of NRBP1 and TSC22Ds on WNK signaling in vitro and in the DCT. TSC22D1.1, TSC22D2, and NRBP1 are localized in DCT WNK bodies, which are cytoplasmic biomolecular condensates associated with WNK activation. In HEK293 cells, long TSC22D isoforms and NRBP1 increase WNK4 activity. DCT-specific NRBP1-knockout mice have reduced NCC phosphorylation and activate a compensatory response. Thus, NRBP1 and long TSC22D proteins are positive modulators of WNK signaling and modulate Na^+^ reabsorption in the kidney. NRBP1 and TSC22Ds likely influence WNK signaling in other tissues, affecting various physiological processes.

## Teaser

The pseudokinase NRBP1 and its associated TSC22D proteins modulate WNK kinases to regulate sodium reabsorption in the kidney.

## INTRODUCTION

The with-no-lysine (K) (WNK) kinase family comprises four members (WNK1-4). These proteins have been implicated in the regulation of cell volume, epithelial electrolyte transport, neuronal intracellular chloride concentration, and cell proliferation (*1*). Most of these functions involve phosphorylation by WNK kinases of their canonical downstream targets Ste20-related proline-alanine-rich kinase (SPAK) and oxidative stress response 1 (OSR1), which, in turn, phosphorylate and modulate the activity of cation chloride cotransporters (CCCs) of the *SLC12* family. Among these CCCs, we find the loop diuretic-sensitive Na^+^:K^+^:Cl^−^ cotransporter (NKCC2) and the thiazide-sensitive Na^+^:Cl^−^ cotransporter (NCC), which mediate NaCl reabsorption in the thick ascending limb and distal convoluted tubule (DCT) of the kidney nephron, respectively.

Mutations in the genes encoding WNK1 and WNK4, among others, are cause of a tubulopathy called familial hyperkalemic hypertension (FHHt) (also known as pseudohypoaldosteronism type II or Gordon syndrome) (*2*–*4*). Patients with FHHt present with hypertension, hyperkalemia, and metabolic acidosis. These electrolytic derangements are essentially due to the overactivity of NCC that is exclusively present in the apical membrane of the DCT (*5*, *6*). Thus, the role of WNK kinases in DCT physiology has been extensively studied. Evidence suggests that WNK4 is the major catalytically active WNK isoform in these cells (*7*–*9*). Moreover, in DCT, the vast majority of *WNK1* mRNA encodes a short isoform [kidney-specific (KS)–WNK1] lacking the kinase catalytic domain (*10*). Consistent with this, NCC activity and phosphorylation in the DCT are completely lost in *Wnk4* knockout (KO) mice (*7*).

We and others have recently described that, during osmotic stress, a condition in which WNK kinases are activated to promote ion influx to the cell through modulation of CCCs, the interaction of WNK1 with nuclear receptor binding protein 1 (NRBP1) and proteins of the transforming growth factor–β–stimulated clone 22 domain (TSC22D) family (*11*, *12*) is stimulated. The latter are known interactors of NRBP1 (*13*). The NRBP family comprises two members (NRBP1 and NRBP2) that are highly homologous. These proteins are pseudokinases that are evolutionarily related to WNK kinases (*12*, *14*). They present a general architecture similar to that of WNK kinases, with a short N-terminal domain, followed by the pseudokinase domain, and a C-terminal domain that is much smaller than that of WNK kinases but that at least contains one common feature, which is a globular conserved C-terminal (CCT) domain (*12*, *15*) [also known as PF2 domain; (*16*–*18*)]. Similar CCT domains are present in WNKs and SPAK/OSR1. The CCT domains of SPAK and OSR1 have been shown to mediate the interaction of these kinases with RFxV motifs present in interacting partners like CCCs and WNKs. In addition, other protein binding motifs have been shown to be present in the C-terminal domain of NRBP1, like a myeloid leukemia factor 1 binding motif (*19*), an Elongin BC binding motif (*20*), and a Cullin binding motif (*21*).

**Fig. 1.**
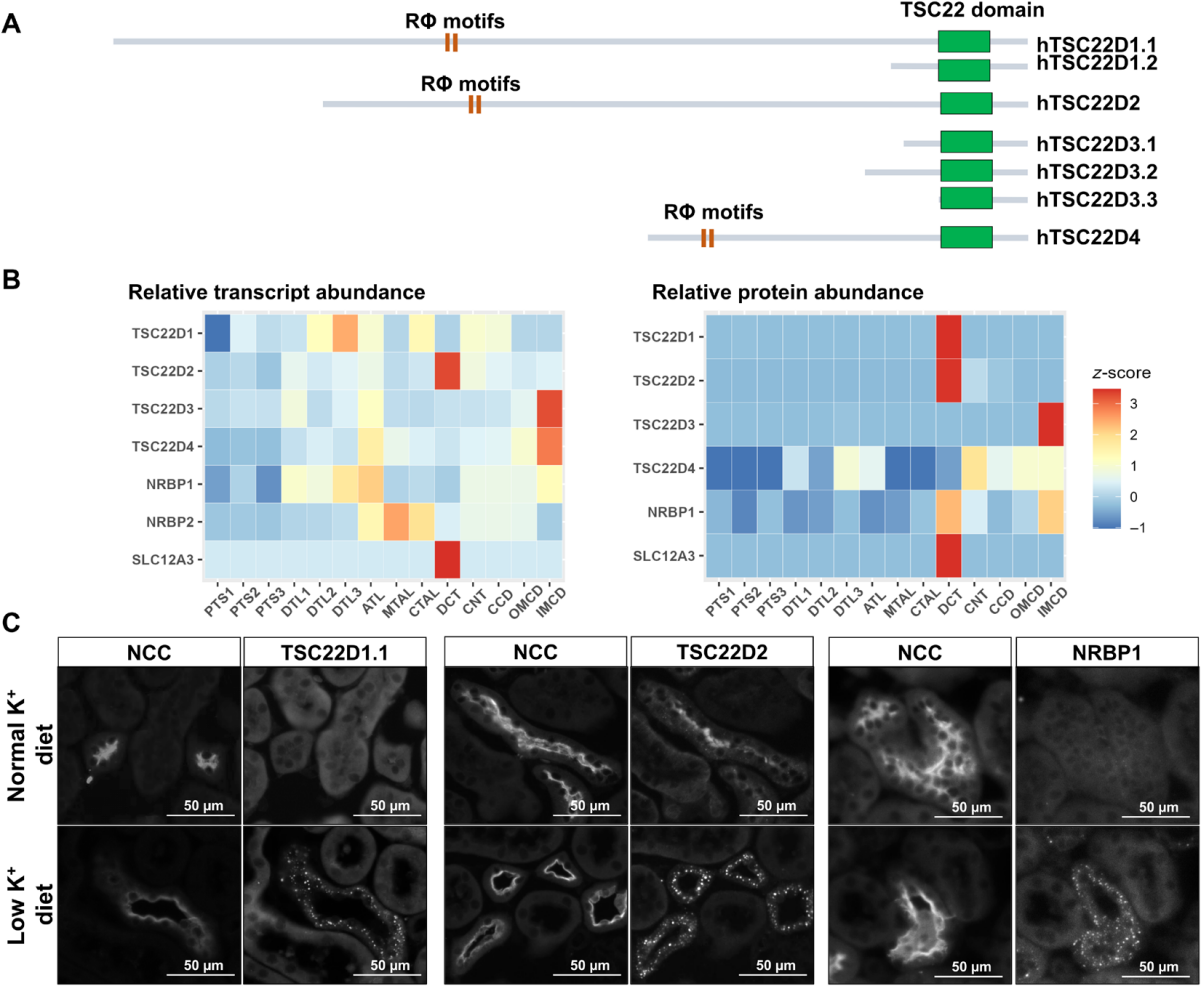
Within the kidney, TSC22D2 and TSC22D1.1 are enriched in DCT cells, and these proteins, as well as NRBP1, are observed in cytoplasmic puncta in DCTs of hypokalemic mice. (**A**) The TSC22D protein family. The four *TSC22D* genes (*TSC22D1-4*) present in mammals can give rise to multiple isoforms through alternative slicing. TSC22D isoforms can be classified into short and long, depending on the absence or presence, respectively, of a long, disordered N-terminal domain. The highly conserved C-terminal domain (named TSC22 domain) is present in all isoforms and is the only region of the protein that presents structural organization. This domain is involved in homo- and heterodimerization (*12*). Only the long isoforms present Rϕ motifs located within a region of intrinsic disorder that have been shown to be necessary for interaction with the CCT domain of NRBP1 but may also mediate interaction with SPAK/OSR1 and WNKs (*11*–*13*). (**B**) Relative abundance of transcripts and proteins of the NRBP and TSC22D families along different renal segments. To the left, the heatmap shows the relative abundance of *Nrbp* and *Tsc22d* transcripts along the 14 mouse nephron segments. Data from single-cell RNA sequencing experiments performed with manually microdissected segments produced by Chen and coworkers was used (*26*). To the right, the heatmap shows the relative abundance of NRBP and TSC22D proteins along the different rat nephron segments. Data from quantitative proteomic experiments performed with manually microdissected nephron segments were used (*27*). *z*-score normalization was applied to the data to standardize the values across each feature. (**C**) Immunofluorescent staining of kidney tissue from C57Bl/6 WT mice on normal or low K^+^ diet with antibodies against TSC22D2, TSC22D1.1 (the long isoform), and NRBP1. DCT cells were identified by NCC staining. Tissue from at least three male or female mice per condition was analyzed.

The TSC22D family is encoded in mammals by four different genes (*TSC22D1-4*), some of which can produce multiple isoforms. Of note, the TSC22D isoforms can be classified into short and long isoforms depending on the length of their N-terminal domain (Fig. 1A). Long isoforms contain a long and mainly disordered N-terminal domain (*11*, *12*), followed by a short, structured C-terminal domain known as TSC22 domain that is implicated in homo- and heterodimerization (*12*). Within the disordered N-terminal domain, a conserved region that mediates interaction with NRBP1 has been identified (*11*, *13*). Within this region, in addition to a canonical RFxV motif, a highly conserved RWxC motif was also identified that participates in the interaction with NRBP1. Thus, it was proposed that CCT-binding motifs could be termed Rϕ (where ϕ represents an hydrophobic residue) (*12*). Short TSC22D isoforms lack most of the disordered N-terminal region and, thus, lack Rϕ motifs but retain the C-terminal TSC22 domain. Previous work has shown that short isoforms exert opposing effects to long isoforms (*13*).

Hyperosmotic stress has been shown to induce the formation of WNK1-containing biomolecular condensates that are formed through liquid-liquid phase separation in response to molecular crowding (*22*). The long, intrinsically disordered C-terminal domain of WNKs was shown to be essential for the formation of WNK condensates. Xiao *et al.* (*11*) recently showed that TSC22D and NRBP proteins localize to WNK1-containing condensates induced by hypertonic stress. Thus, the term TWN (TSC22, WNK, and NRBP1) bodies was proposed to refer to these condensates. Moreover, it was shown, through BioID proximity ligation assays, that, under hyperosmotic conditions, an extensive set of physical associations among TSC22D1-4, WNK1-3, and NRBP1-2 are enhanced. Last, like for WNK1, it was shown that NRBP1 and TSC22D proteins are also essential for adequate cell volume regulation in response to osmotic stress (*11*). In the kidney and, specifically, in DCT cells, WNK condensates are observed under certain conditions, in which WNK kinase abundance increases (e.g., hypokalemia and FHHt) (*23*–*25*).

Data available in kidney proteomic and transcriptomic databases show that *TSC22D2* mRNA and TSC22D2 protein abundances are considerably higher in DCT than in other nephron segments (Fig. 1B) (*26*, *27*). The data also reveal that the long isoform of *TSC22D1* (TSC22D1.1) is also highly enriched within the DCT (*26*). In addition, KO of the *Tsc22d3* gene in mice (which encodes several short isoforms) leads to NCC overactivation and an FHHt-like phenotype (*28*). TSC22D3, also known as glucocorticoid-induced leucine zipper, has been shown to reduce SPAK and NCC phosphorylation when overexpressed in cells. These observations suggest that proteins from the TSC22D family in conjunction with NRBP may play a relevant role in WNK signaling and DCT physiology. In the present study, we explored the localization of specific TSC22Ds and NRBP1 in kidney tissues and investigated how these proteins affect the WNK4-SPAK/OSR1 pathway. Our data strongly suggest a physiological role of these proteins in DCT, and this role is most likely generalizable to other cell types and tissues where WNK function is essential.

## RESULTS

### Expression of the long TSC22D isoforms TSC22D2 and TSC22D1.1 is enriched in the DCT where these proteins are present in WNK bodies together with NRBP1

**Fig. 2.**
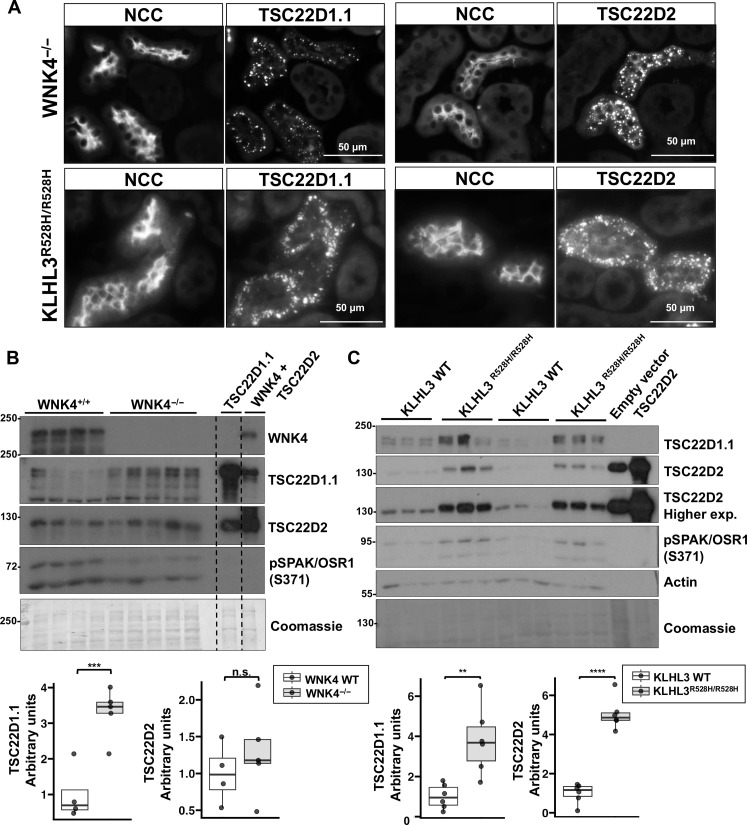
TSC22D2 and TSC22D1.1 are observed in cytoplasmic puncta in the DCTs of WNK4^−/−^ and KLHL3^R528H/R528H^ mice. (**A**) Immunofluorescent staining of kidney tissue from *Wnk4*^−/−^ (top panels) or *Klhl3*^R528H/R528H^ (bottom panels) mice maintained on regular chow. TSC22D2- and TSC22D1.1-positive cytoplasmic puncta were observed almost exclusively in NCC-positive cells. Staining of tissue from at least three male or female mice per condition was performed with similar results in all cases. (**B**) Immunoblots performed with kidney samples from male homozygous *Wnk4* KO mice and their WT littermates. The last two lanes were loaded with lysates from HEK293 cells transfected with TSC22D1.1 and TSC22D2 as control. Results of quantitation are presented in the graphs below. (**C**) Immunoblots performed with kidney samples from male homozygous *Klhl3*-R528H mice and their WT littermates. The last two lanes were loaded with lysates from HEK293 cells transfected with empty vector or TSC22D2 as control. Densitometric values were analyzed by unpaired Student’s *t* test. Quantitative analysis is represented in box plots showing median and interquartile range. **P < 0.01; ***P < 0.001; ****P < 0.0001.

A particular feature of the DCT is that, under certain conditions, WNK4, KS-WNK1, SPAK, and OSR1 are observed to localize in cytoplasmic puncta (*23*–*25*) that are believed to comprise biomolecular condensates similar to the ones formed in cells treated with hypertonic conditions (*22*). These condensates have been termed WNK bodies. Thus, we sought to investigate the distribution of TSC22D2, TSC22D1.1, and NRBP1 proteins in kidney tissue by immunofluorescent staining at baseline and in mice maintained on a low-K^+^ diet (LKD), a condition in which WNK bodies and NCC activation are observed (*23*, *25*). At baseline, no clear positive signal was observed with the TSC22D1.1 and NRBP1 antibodies, whereas the TSC22D2 antibody displayed a clear apical signal that was exclusively detected in NCC-positive cells (i.e., DCT cells) (Fig. 1C). In contrast, in kidneys from mice on LKD, the three antibodies gave a similar signal: a cytoplasmic punctuate signal. This signal was mainly observed in DCT cells (Fig. 1C) but was also seen in a few sporadic NCC-negative cells (fig. S1). The latter were presumably CNT cells in which KS-WNK1 expression, a major driver of WNK body formation, is also observed (*10*, *23*). We cannot rule out that the absence of signal observed for NRBP1 and TSC22D1.1 in baseline conditions may have been due to insufficient sensitivity of the antibody assay, as NRBP1 expression, for example, is presumed to be ubiquitous and constitutive. However, detection within WNK bodies is likely to be made possible as a result of a high concentration of these proteins within these structures making these more readily detected in the immunofluorescence experiments. A punctate cytoplasmic localization for TSC22D1.1 and TSC22D2 in DCT cells was also observed in other mouse models in which large WNK bodies have been previously reported: *Wnk*4 KO mice (*25*) and *Klhl3*-R528H knock-in mice, a model of FHHt in which WNK4 and KS-WNK1 abundance in the DCT is increased (*24*) (Fig. 2A). Greater TSC22D1.1 and TSC22D2 abundance was observed in *Klhl3*-R528H knock-in mice, and greater abundance of TSC22D1 was also observed in *Wnk4* KO mice (Fig. 2, B and C). In this latter model, WNK bodies’ presence is thought to be part of a compensatory response. Despite being described as transcriptional factors, we failed to detect nuclear signal of TSC22D1.1 or TSC22D2 in both transgenic models where the abundance of these proteins was increased.

**Fig. 3.**
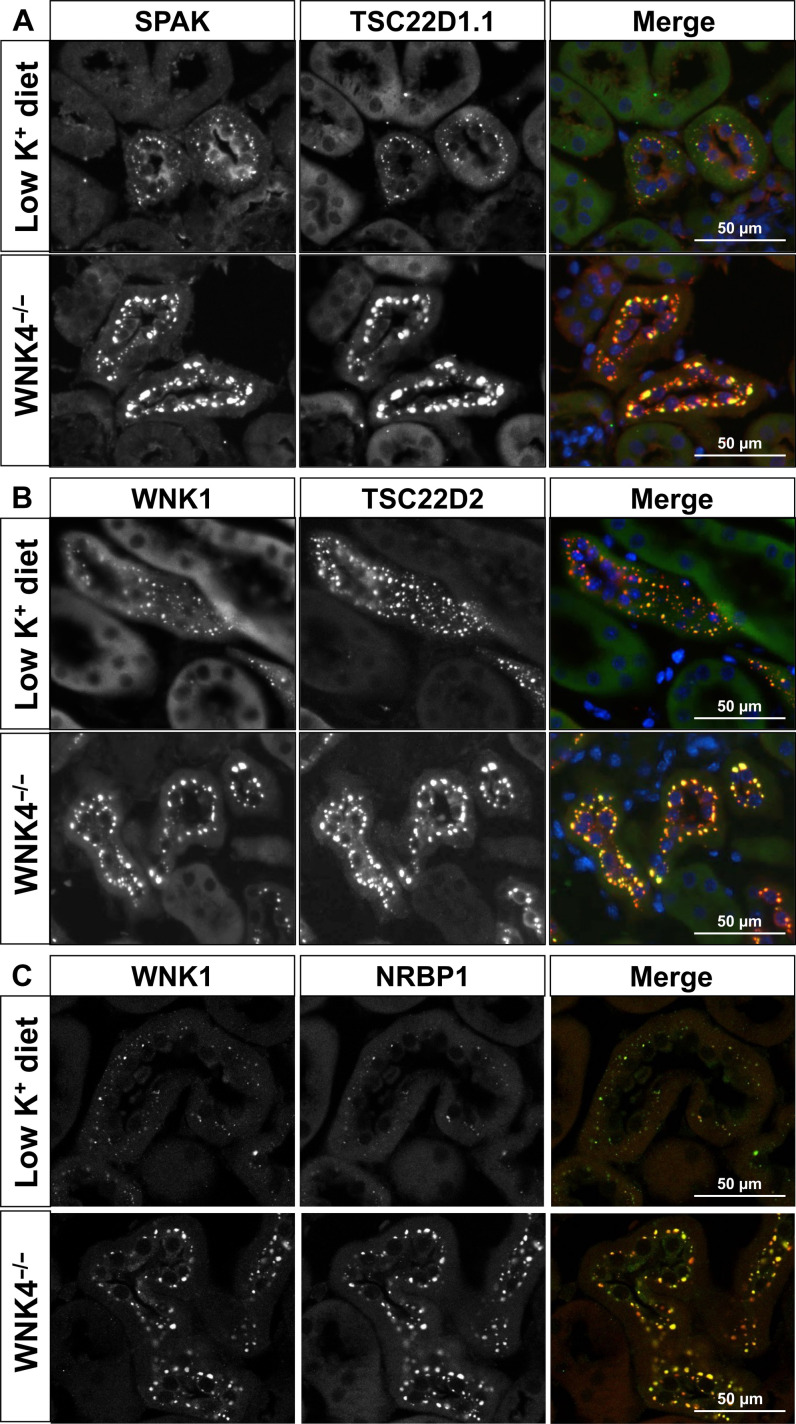
TSC22D2-, TSC22D1.1-, and NRBP1-positive cytoplasmic puncta are WNK bodies. Immunofluorescent staining of kidney tissue from C57Bl/6 WT mice maintained on LKD and *Wnk4*^−/−^ on regular chow. Costaining of TSC22D1.1 with SPAK (**A**), TSC22D2 with WNK1 (**B**), or NRBP1 with WNK1 (**C**) confirmed that TSC22D2-, TSC22D1.1-, and NRBP1-positive cytoplasmic puncta observed in DCT cells correspond to WNK bodies.

We confirmed that TSC22D1.1, TSC22D2, and NRBP1-positive cytoplasmic puncta correspond to WNK bodies through colocalization with SPAK or WNK1, which was observed in tissues from mice on LKD and from *Wnk4*^−/−^ mice (Fig. 3). The signal observed with the WNK1 antibody most likely corresponded to KS-WNK1 (*24*, *29*). Together, these results show that TSD22D1.1 and TSC22D2 within the kidney are mainly expressed in the DCT where these proteins colocalize with elements of the WNK-SPAK/OSR1 pathway in conditions in which WNK bodies are formed. In addition, NRBP1 is also present in DCT WNK bodies.

### Coexpression of NRBP1 with long TSC22D isoforms promotes activation of the WNK4-SPAK pathway

**Fig. 4.**
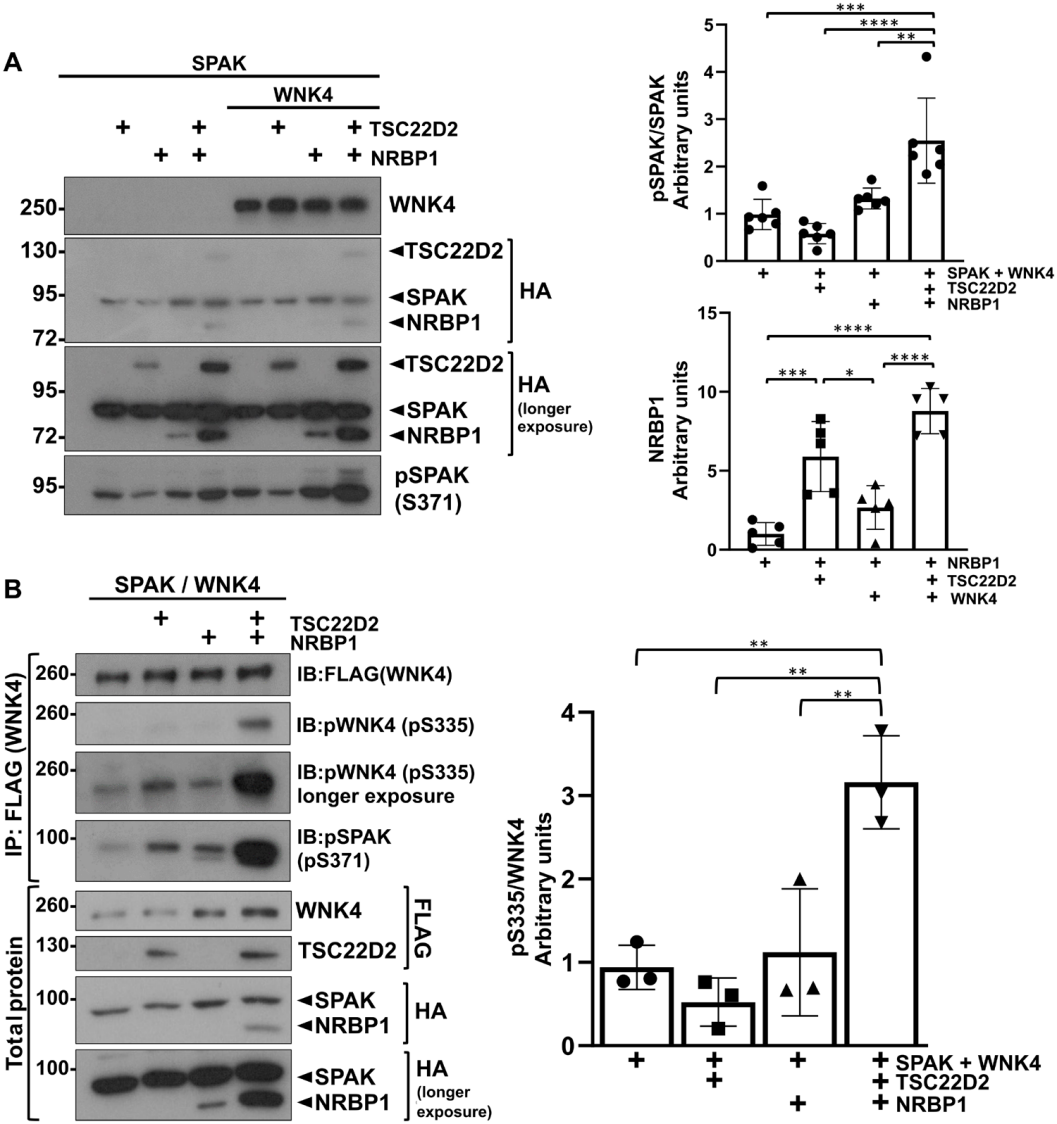
NRBP1 acts in concert with TSC22D2 to promote WNK4 activation. (**A**) The effect of coexpression of NRBP1 with TSC22D2 on WNK4’s ability to phosphorylate SPAK was assessed in HEK293 cells. Cells were transiently transfected with SPAK-HA, WNK4-FLAG, NRBP1-HA, and TSC22D-HA as indicated. Forty-eight hours posttransfection, immunoblots were performed to confirm expression of transfected proteins and to analyze phosphorylation levels of SPAK (pSPAK-S371, which corresponds to the S motif site, previously identified as S373; see Q9UEW8 in UniProt). An increase in pSPAK was observed upon coexpression of NRBP1 and TSC22D2 in the presence of WNK4. Results of quantitation are shown in the graphs to the right showing means ± SD. Analysis of variance (ANOVA) followed by Tukey’s post hoc tests were performed to identify statistically significant differences. *P < 0.05; **P < 0.01; ***P < 0.001; ****P < 0.0001. At least three independent experiments were performed. (**B**) *WNK1* KO HEK293 cells were transiently transfected with WNK4-FLAG and SPAK-HA, as well as TSC22D2-FLAG, NRBP1-HA or both. This cell line was used to reduce transautophosphorylation of WNK4 by endogenous WNK1. Forty-eight hours posttransfection, autophosphorylation levels of WNK4 at the S335 activation site were assessed through immunoblot (IB) of immunoprecipitated (IP) WNK4 using the pWNK1-S382 antibody that has been shown to cross-react with pWNK4 (*46*). A robust increase in WNK4-S335 phosphorylation was observed in cells coexpressing NRBP1 and TSC22D2. Results of quantitation are shown in the graph to the right showing means ± SD. ANOVA followed by Tukey’s post hoc tests were performed to identify statistically significant differences. **P < 0.01. At least three independent experiments were performed.

To explore the physiological role of TSC22D and NRBP proteins in the DCT, we decided to study the effect of these proteins on the activity of the WNK4-SPAK pathway. We focused on WNK4 because, as mentioned earlier, this is the main (and probably the only) catalytically active WNK kinase present in the DCT. Human embryonic kidney (HEK) 293 cells were cotransfected with SPAK, WNK4, NRBP1, and long TSC22D isoforms in different combinations (Fig. 4A and fig. S2). Levels of SPAK phosphorylation (pSPAK) were assessed by immunoblot as a readout for WNK pathway activity. In the absence of transfected WNK4, TSC22D1, and TSC22D4, when expressed together with NRBP1, increased the levels of pSPAK (fig. S2). A trend toward increased pSPAK was also observed in the presence of TSC22D2 and NRBP1. In the presence of WNK4, all three long TSCs—TSC22D1.1, TSC22D2 and TSC22D4—had an activating effect on SPAK phosphorylation when expressed in conjunction with NRBP1 (Fig. 4A and fig. S2). Of note, activation of WNK4-SPAK was also observed in the presence of the NRBP1 paralogue NRBP2 when coexpressed with TSC22D2 (fig. S3).

In these experiments, we noted that expression of long TSC22D proteins or WNK4 increased the amount of NRBP1 protein. This effect was additive, as higher NRBP1 levels were observed in the presence of a TSC22D and WNK4 than in the presence of either one of these proteins alone. The increase in pSPAK levels in the TSC22 + NRBP1 groups was not due to the higher abundance of NRBP1 in this group because, when we compared the effect of increasing NRBP1 abundance by increasing the amount of transfected DNA with the effect observed upon coexpression of TSC22D2, a much more notable increase in pSPAK was observed in the latter case (fig. S4).

Last, to investigate whether the observed increase in pSPAK levels was due to increased activity of the WNK kinase (either the endogenous WNK or the transfected WNK4), we assessed WNK4 phosphorylation at the T-loop site [S335 in human WNK4; this is a site that is autophosphorylated and whose phosphorylation promotes kinase activation; (*30*)]. A clear increase in WNK4 T-loop phosphorylation was observed in the presence of TSC22D2 and NRBP1 (Fig. 4B), suggesting that these proteins increase the ability of WNKs to autophosphorylate. These findings are consistent with the observations made by us in a parallel work in which NRBP1 was shown to increase activity of WNK4 in in vitro kinase assays (*12*).

### KO of NRBP1 in the DCT reduces NCC phosphorylation

**Fig. 5.**
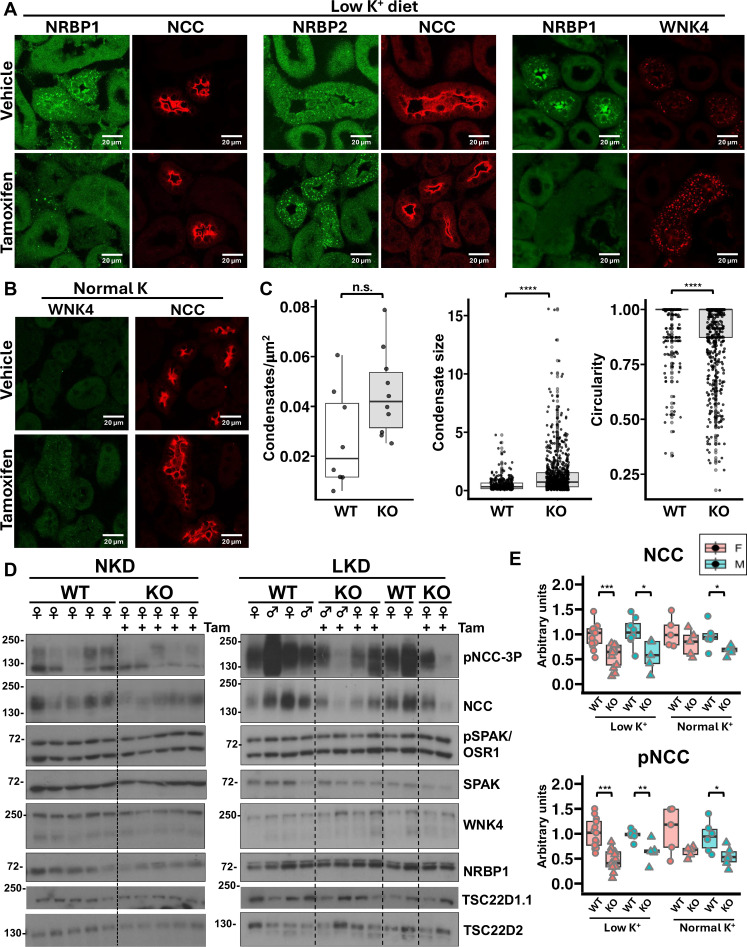
DCT-specific *Nrbp1* KO mice have decreased NCC phosphorylation. (**A**) Absence of NRBP1 in the DCT was confirmed by immunofluorescent staining of kidney sections from mice on LKD (left panels). NCC-positive tubules from KO mice had no NRBP1-positive cytoplasmic condensates and the diffuse cytoplasmic signal was less intense than in other cell types. NRBP2 immunofluorescent staining revealed that NRBP2 is also present in DCT condensates from WT mice on LKD (middle panels). In KO mice, NRBP2-positive condensates appeared to be larger. WNK4-positive cytoplasmic condensates in DCT cells from *Nrbp1* KO mice also appeared to be larger. (**B**) Immunofluorescent staining of WNK4 and NCC in WT and *Nrbp1* KO mice maintained on NKD. No clear positive signal was observed for NRBP1 in WT mice under this condition, whereas small WNK bodies were observed in DCTs of tamoxifen-treated KO mice. (**C**) Analysis of number (left), size (middle), and circularity (left) of WNK4-positive WNK bodies observed in WT and DCT-specific KO mice was performed in ImageJ, and results are represented in box plots showing median and interquartile range. ****P < 0.0001. Non-parametric Wilcoxon tests were performed to identify statistically significant differences. n.s., not significant. (**D**) Immunoblots performed with kidney protein lysates from WT and DCT-specific *Nrbp1* KO mice. Results of quantitation of NCC and pNCC blots are presented in (**E**). Results of quantitation are represented in box plots showing median and interquartile range. Student’s *t* tests were performed to identify statistically significant differences. *P < 0.5; **P < 0.01; ***P < 0.001.

To confirm the role of NRBP1/TSC22Ds in the modulation of the WNK4-SPAK pathway in vivo, we generated DCT-specific, inducible *Nrbp1* KO mice by crossing conditional-ready *Nrbp1* mice [European Mouse Mutant Archive (EMMA) strain ID: 09610] with NCC-Cre mice (*31*). Mice were studied on a normal-K^+^ diet (NKD) and on a LKD for 5 days, the latter to promote WNK4-SPAK pathway activation in the DCT and the formation of WNK bodies. NRBP1 immunostaining of tissues from mice under LKD confirmed the absence of NRBP1 in DCTs of tamoxifen-treated mice (Fig. 5A). NRBP1-positive condensates were observed in sporadic cells that were NCC negative, supporting the specificity of the cell-type–specific targeting strategy (fig. S5). In contrast, NRBP2 staining in KO mice on LKD showed a clear positive signal in the DCT, where NRBP2 localized in WNK bodies. Large WNK4-positive WNK bodies were also observed in the KOs. Moreover, under NKD WNK bodies were observed in the KO mice, whereas, under these conditions, WNK bodies were absent in the control mice (Fig. 5B). Analysis of WNK4-positive WNK bodies of mice under LKD showed that these were larger in size and had reduced circularity in the KO mice (Fig. 5C and fig. S6). A tendency for higher number of WNK bodies was also observed. Reduced pNCC and NCC levels were observed in *Nrbp1* KOs (Fig. 5, D and E). Previous work has also shown in WNK4 and other WNK-pathway deficient mice that loss of NCC phosphorylation results in a marked reduction in NCC protein (*7*, *32*). No differences in the levels of pSPAK/OSR1, SPAK, NRBP1, or WNK4 were observed in the blots presumably due the ubiquitous nature of these proteins that may have obscured changes occurring only in the DCT. However, we did observe reduced pSPAK signal in the apical membrane of mice on LKD by immunofluorescent staining (fig. S6), with no differences in apical SPAK signal. Also, larger pSPAK-positive condensates were observed in *Nrbp1* KO mice. Thus, the decreased pSPAK apical signal could be due to decreased total pSPAK abundance within DCT cells, but the different staining pattern observed between genotypes makes quantification of the latter impossible.

In conclusion, the presence of NRBP2 in the DCT and the likely compensatory increase in WNK bodies observed in the KOs did not appear to be sufficient to prevent a defect in pathway activation in the absence of NRBP1. This compensation, however, probably prevented a more marked decrease in NCC phosphorylation and activity which is why no marked electrolytic derangements were observed other than a tendency to hypokalemia [table S1; female wild type (WT): 2.68 ± 0.6 mequiv/liter versus female KO: 2.37 ± 0.47 mequiv/liter, *P* = 0.141; male WT: 2.69 ± 0.6 mequiv/liter versus male KO: 2.17 ± 0.5 mequiv/liter, *P* = 0.055].

### Activation of the WNK4-SPAK pathway by NRBP1/TSC22D2 depends on direct or indirect interactions mediated by CCT domains and RΦ motifs

**Fig. 6.**
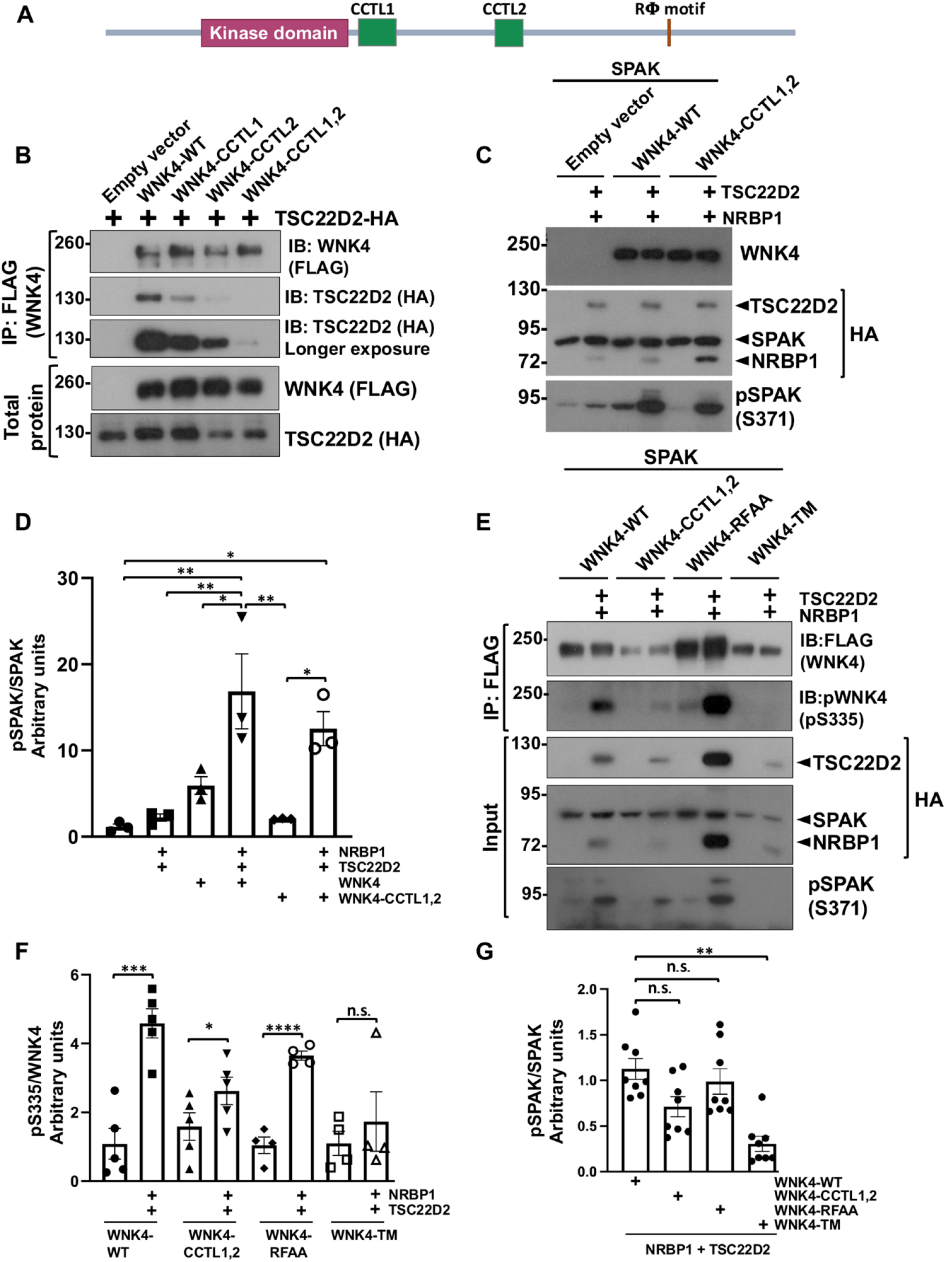
The activator effect of NRBP1/TSC22D2 on WNK4 depends on direct or indirect interactions mediated by CCT domains and RFxV motifs. (**A**) Primary structure of WNK4 showing the location of CCTL domains and Rϕ motif. (**B**) *WNK1* KO HEK293 cells were transiently transfected with TSC22D2-HA and WT WNK4-FLAG or WNK4 mutants in which key residues within the first (CCTL1, F476A/F478A), second (CCTL2, F703A/F705A), or both (CCTL1,2) CCTL domains were mutated. The levels of coimmunoprecipitated TSC22D2-HA with WNK4-FLAG were assessed. (**C**) *WNK1* KO HEK293 cells were transiently transfected with SPAK-HA and WNK4-FLAG-WT or WNK4-FLAG-CCTL1,2. Where indicated, NRBP1-HA and TSC22D2-HA were cotransfected to assess their effect on WNK4-mediated SPAK phosphorylation. Despite the decreased TSC22D2–WNK4-CCTL1,2 binding observed in (B), activation of WNK4-mediated SPAK phosphorylation by NRBP1/TSC22D2 was observed in the presence of this mutant. (**D**) Results are means ± SD. (**E**) *WNK1* KO HEK293 cells were transiently transfected with SPAK-HA and FLAG-tagged WNK4-WT, WNK4-CCTL1,2, WNK4-RFAA [with mutated RFXV motif necessary for direct SPAK binding (*16*), (R1016A, F1017A)], or the triple mutant (WNK4-TM) containing the CCTL1,2 and the RFAA mutations. Where indicated, NRBP1-HA and TSC22D2-HA were also cotransfected to assess their effect on WNK4 T-loop autophosphorylation on immunoprecipitated WNK4. (**F**) Results of quantitation (means ± SD). No increase in pWNK4-S335 was observed upon NRBP1/TSC22D2 coexpression in the presence of WNK4-TM, suggesting that the activating effect observed in (C) in the presence of the WNK4-CCTL1,2 mutant was indirectly facilitated by SPAK. (**G**) Quantitation of phosphorylated SPAK in the presence of NRBP1 and TSC22D2. Blots in which no signal was observed in the WNK4-TM group were not included in the analysis due to the impossibility to quantify. ANOVA followed by Tukey’s post hoc tests were performed. *P < 0.05; **P < 0.01; ***P < 0.001; ****P < 0.0001. At least three independent experiments were performed. n.s., not significant.

It is well-known that the direct WNK-SPAK/OSR1 interaction depends on the CCT domain in SPAK/OSR1 and the Rϕ motifs present in WNKs (*16*, *33*, *34*). WNK kinases, in addition to Rϕ motifs, also contain two CCT-like (CCTL) domains termed CCTL1 and CCTL2 (Fig. 6A), whose function has remained elusive (*16*, *35*). The key residues within the CCT domain of OSR1 that establish interactions with residues of Rϕ motifs have been characterized (*18*). We have previously shown that mutations of some of these key residues in the CCTL domains of WNK4 (F476A and F478A) and (V701A and F703A) reduce the ability of the kinase to phosphorylate SPAK, without affecting its interaction with SPAK or its homo- or heterodimerization (*16*). Thus, we hypothesized that these CCTL domains of WNKs may be relevant for the interaction with the Rϕ motifs of long TSC22D proteins and that a reduced interaction with these proteins may explain the decreased activity of these mutants. To test this, we performed immunoprecipitation experiments in which we observed that mutations within the first (CCTL1) or second (CCTL2) CCT motifs of WNK4 reduced its interaction with TSC22D2 and that mutations within both domains (CCTL1,2 mutant) further reduced the interaction (Fig. 6B).

To determine whether the reduced activity of the WNK4 CCTL1,2 mutant was due to reduced interaction with TSC22D proteins, we examined the effect of NRBP1 and TSC22D2 coexpression on SPAK phosphorylation in the presence of this mutant version of WNK4. Unexpectedly, coexpression with NRBP1 and TSC22D2 substantially enhanced the mutant’s ability to phosphorylate SPAK (Fig. 6C). This led us to hypothesize that NRBP1 and TSC22D2 might activate WNK4-CCTL1,2 through an indirect interaction mediated by SPAK (i.e., TSC22D2 may recruit WNK4 through binding to SPAK). This was suspected given that the absence of colocalization of TSC22D2 with the CCTL1,2 mutant was rescued in the presence of SPAK (fig. S7). To test this hypothesis, we assessed WNK4 activation by measuring WNK4-T-loop phosphorylation in the WT protein, the WNK4-CCTL1,2 mutant, the WNK4-RFAA mutant (in which SPAK interaction is abrogated by mutating the Rϕ motif that mediates interaction with SPAK), and a triple mutant (TM) called WNK4-TM, in which both CCTL domains and the SPAK-binding site were mutated. This latter mutant was, thus, completely unable to establish CCT-Rϕ interactions. We observed that NRBP1 and TSC22D2 increased the level of T-loop phosphorylation for WNK4-WT, WNK4-RFAA, and WNK4-CCTL1,2, but this was not observed with the WNK4-TM construct (Fig. 6C). This suggests that the activation of the WNK4-CCTL1,2 mutant was facilitated through an indirect interaction with NRBP1 and TSC22D2 mediated by SPAK. In addition, stimulation of SPAK phosphorylation was also observed in the presence of all WNK mutants, with exception of the WNK4-TM (Fig. 6E), thus suggesting that SPAK phosphorylation can occur in the absence of canonical SPAK-CCT WNK4-RFxV binding, which is consistent with the observation that SPAK can be recruited to condensates containing the WNK4-RFAA mutant, probably through TSC22D binding. In summary, it would appear that there is a very large combinatory of Rϕ-CCT interactions in this pathway’s components that could partially compensate for the absence of a CCT domain or Rϕ motif in any single protein.

### Both WNK proteins and long TSC22D proteins can promote condensate formation when overexpressed in cultured mammalian cells

**Fig. 7.**
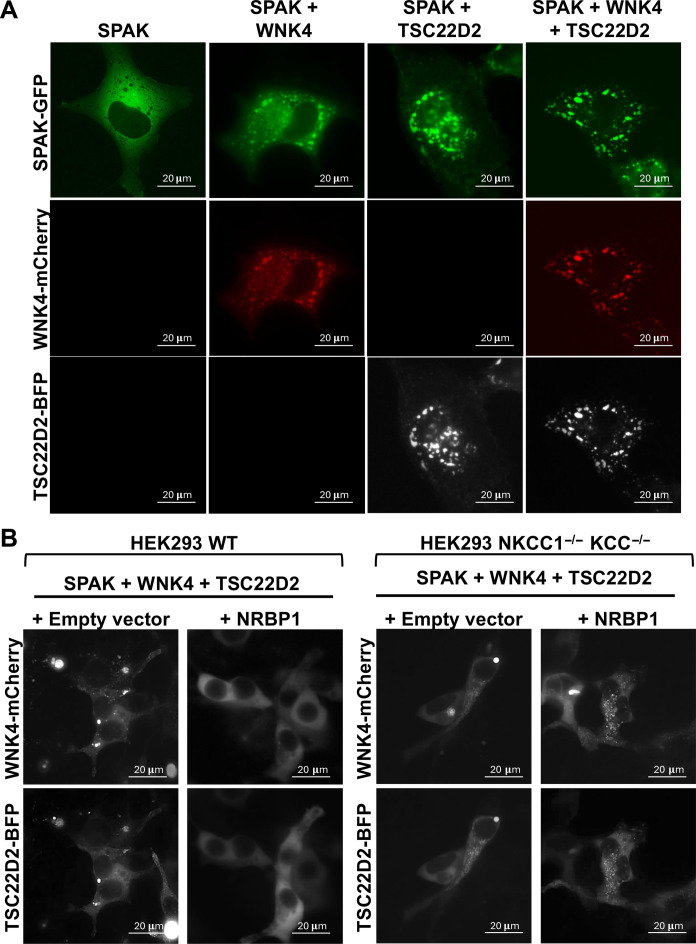
WNK4 and TSC22D2 expression in cultured cells promotes condensate formation and NRBP1 coexpression prevents condensate formation through an effect on cell volume. (**A**) COS7 cells were transfected with GFP-tagged SPAK and the indicated constructs. When SPAK-GFP was transfected alone, a diffuse cytoplasmic distribution was observed. In contrast, SPAK-containing cytoplasmic condensates were observed in the presence of overexpressed WNK4 or TSC22D2, and larger condensates were observed in the presence of both WNK4 and TSC22D2. (**B**) When WNK4-mCherry, SPAK-GFP, and TSC22D2-BFP were transfected in WT HEK293 cells, WNK4/TSC22D2-positive condensates were observed. However, when NRBP1 was also transfected, no condensates were observed, and cells had a more swollen appearance. In contrast, in *SLC12* KO cells (validated in fig. S9), condensates were observed in cells overexpressing the four proteins (i.e., including NRBP1), suggesting that the prevention of condensate formation observed in the presence of NRBP1 in HEK293 WT cells was secondary to an effect on transport and most likely cell volume.

When we observed subcellular localization of a green fluorescent protein (GFP)–tagged SPAK we noticed a diffuse cytoplasmic localization in the absence of transfected WNK4 and TSC22D2. However, in the presence of WNK4 or TSC22D2, SPAK-GFP localized to cytoplasmic condensates. These condensates became more prominent when TSC22D2 and WNK4 were coexpressed (Fig. 7A). This suggests that WNK4 and TSC22D2 overexpression can promote condensate formation that is consistent with the fact that these proteins contain large intrinsically disordered domains (*11*, *12*). Nevertheless, it should be mentioned that these results must be taken with caution, given that the overexpression of proteins may per se induce their condensation. However, these condensates show sensitivity to changes in extracellular tonicity as observed for endogenous condensates, suggesting that their formation is subject to similar regulatory mechanisms (fig. S8 and movies S1 and S2) (*22*). No condensates were observed when SPAK, WNK4, and TSC22D2 were coexpressed with NRBP1 (Fig. 7B). Given that increased pSPAK levels are observed under this condition (Fig. 4) and given that the cells transfected with the four constructs had a more swollen appearance than cells transfected without NRBP1 (Fig. 7B), we suspected that absence of condensation could be due to an SLC12 cotransporter-mediated increase in cell volume that would decrease molecular crowding and prevent WNK condensation. This hypothesis was supported by the observation that, in *SLC12* KO cells, in which NKCC1 and all KCCs were knocked out and absence of SLC12 activity was confirmed (fig. S9), NRBP1 overexpression in the presence of SPAK, WNK4, and TSC22D2 did not prevent condensate formation (Fig. 7B).

### Colocalization of WNKs and TSC22D proteins in condensates depend on both direct and indirect interactions

**Fig. 8.**
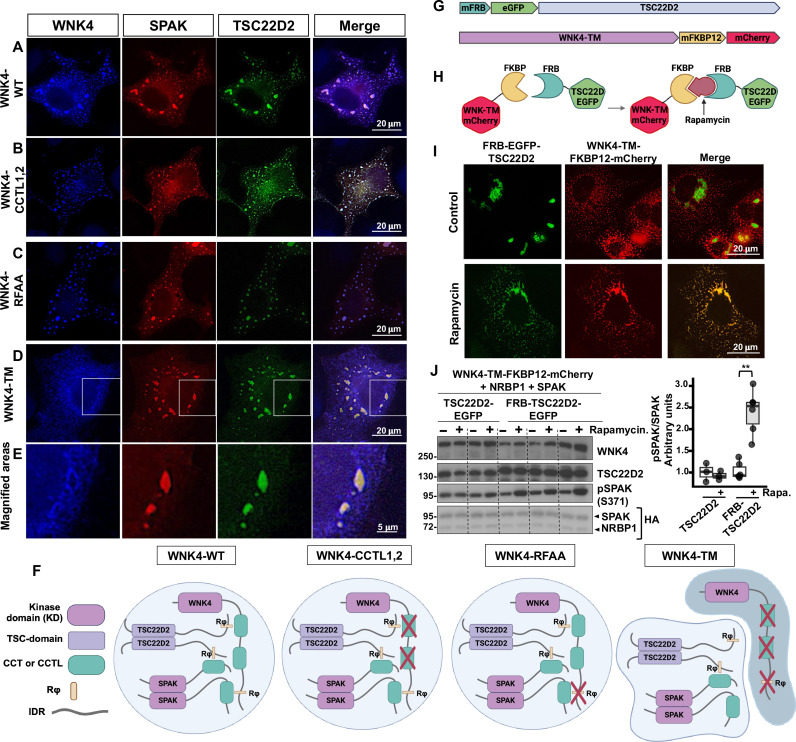
Colocalization of TSC22D2, WNK4, and SPAK within biomolecular condensates promotes pathway activation. COS7 cells were transiently transfected with SPAK-mCherry, TSC22D2-GFP, and the indicated WNK4 constructs (**A** to **D**). Subcellular localization of WNK4 was observed by immunostaining with an anti-WNK4 antibody. Colocalization of WNK4 with TSC22D2 and SPAK in cytoplasmic condensates was observed with the WT WNK4 (A) and was not affected by mutation of both CCTL domains (WNK4-CCTL1,2) (B) or by mutation of the Rϕ motif of WNK4 (WNK4-RFAA) (C). However, when the three regions were mutated (WNK4-TM), mutually exclusive WNK4-containing condensates, as well as SPAK-TSC22D2-containing condensates were observed within cells (D and E). (**E**) Magnification of the areas delimited with white boxes. (**F**) Cartoon summarizing the observations made in (A) to (E). WNK4 can interact with SPAK and TSC22D2 through its Rϕ motif and CCTL domains, respectively. When the CCTL domains of WNK4 are mutated, TSC22D can be recruited to WNK4-containing condensates via SPAK. When the Rϕ motif of WNK4 is mutated, SPAK can be recruited to WNK4-condensates via TSC22D2. When all regions are mutated (WNK4-TM), neither SPAK nor TSC22D2 can be recruited to WNK4 condensates, and independent TSC22D-SPAK containing condensates are formed. Thus, the WNK4-TM construct cannot be activated by NRBP1/TSC222D2 (Fig. 6D). (**G**) Cartoon showing the mFRB-eGFP-TSC22D2 and WNK4-TM-mFKBP-mCherry constructs generated for the experiments presented in (H) and (I). (**H**) The WNK4-TM-mFKBP-mCherry protein is not expected to interact with mFRB-eGFP-TSC22D2 unless rapamycin is present. (**I**) Subcellular distribution of mFRB-eGFP-TSC22D2 and WNK4-TM-mFKBP-mCherry in the absence and presence of rapamycin in COS7 cells. (**J**) Levels of pSPAK in the absence or presence of rapamycin (1 μM) were assessed by immunoblot. Results of quantitation are shown as box plots. Student’s *t* test was performed. **P < 0.01, *n* = 5 from three independent experiments. (G), (H), and (F) were created in BioRender: M. Castaneda bueno (2024), https://BioRender.com/e10z770 and https://BioRender.com/u96f731.

As mentioned above, the WNK4 T-loop phosphorylation experiments shown in Fig. 6E suggested the occurrence of an indirect interaction between WNK4 and TSC22D2 mediated by SPAK. Further supporting this observation, we observed colocalization of WNK4-WT, WNK4-CCTL1,2, and WNK4-RFAA with TSC22D2 and SPAK (Fig. 8, A to C, and fig. S10), but we did not observe colocalization of WNK4-TM with these proteins (Fig. 8, D and E, and fig. S10). In this latter case, we observed WNK4-positive condensates that were negative for TSC22D2 and SPAK, as well as TSC22D2/SPAK-positive condensates that were negative for WNK4 coexisting within cells (Fig. 8E). These results showed that, as long as WNK4 can establish interactions with TSC22D2 or SPAK, it can be recruited to TSC22D2 containing condensates (Fig. 8F). This also suggested that SPAK can maintain indirect interactions with WNK kinases through long TSC22D proteins. Together, these results suggest that any interaction that allows for recruitment of the necessary components to the condensates may be enough to facilitate the activation of this system. This conclusion was further supported by the following experiment. When the WNK4-TM was fused to FK506-binding protein 12 (FKBP12) and TSC22D2 was fused to the FKBP-rapamycin binding (FRB) domain of (mammalian target of rapamycin (mTOR), addition of rapamycin, a drug that induces the FKBP12-FRB interaction, induced colocalization of WNK4-TM and TSC22D2, as well as SPAK activation (Fig. 8, G to J, and fig. S10).

### A short TSC22D isoform, TSC22D3.1, inhibits SPAK phosphorylation

**Fig. 9.**
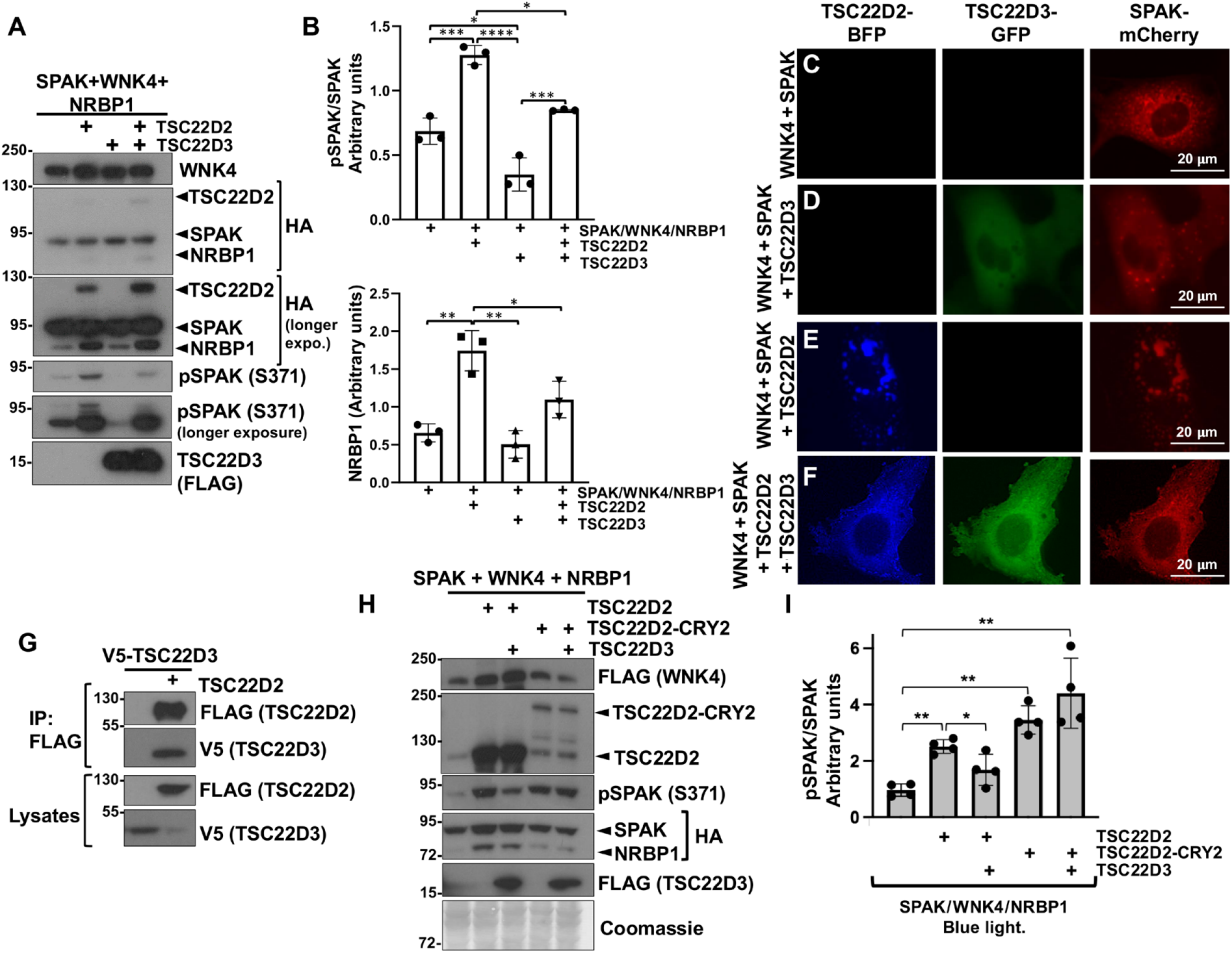
TSC22D3.1, which is a short TSC22D protein, exerts an inhibitory effect on the WNK-SPAK pathway. (**A**) HEK293 cells were transiently transfected with SPAK-HA, WNK4-FLAG, NRBP1-HA, TSC22D2-HA, or TSC22D3-FLAG as indicated. Forty-eight hours posttransfection, pSPAK levels were analyzed by immunoblot. (**B**) Results of quantitation of blots represented in (A) are shown as means ± SD. ANOVA followed by Tukey’s post hoc tests was performed to identify statistically significant differences. *P < 0.05; **P < 0.01; ***P < 0.001; ****P < 0.0001. Three independent experiments were performed. (**C** to **F**) Microscopic images showing cellular distribution of TSC22D2-BFP, TSC22D3-GFP, and SPAK-mCherry. COS7 cells were transfected with the constructs indicated to the left. Coexpression of SPAK with TSC22D3, TSC22D2, or both affects the localization of SPAK within the cell. TSC22D2 induces the formation of larger and more irregular condensates, whereas addition of TSC22D3 reduces the size of condensates and favors a diffuse cytoplasmic localization of SPAK. (**G**) Coimmunoprecipitation assays performed with lysates from HEK293 cells transfected with the indicated constructs. FLAG-tagged TSC22D2 was immunoprecipitated and binding of TSC22D3-V5 was assessed by immunoblot. (**H**) TSC22D2 self-oligomerization prevents the inhibitory effect of TSC22D3. A TSC22D2-Cry2 fusion construct was used for these experiments. The Cry2-Clust domain fused to TSC22D2 is known to oligomerize in response to stimulation with blue light. HEK293 cells were transfected with the indicated constructs. Forty-eight hours after transfection, cells were stimulated with blue light for 30 min and then lysed. SPAK phosphorylation levels were assessed by immunoblot. (**I**) Results of quantitation of blots presented in (H) are shown as are means ± SD. ANOVA followed by Tukey’s post hoc tests were performed to identify statistically significant differences. *P < 0.05; **P < 0.01. At least three independent experiments were performed.

Short TSC22D isoforms have been previously shown to exert opposite effects to TSC22D long isoforms (*13*). Moreover, KO of *Tsc22d3* in mice (which encodes for several short TSC22D isoforms) produces an FHHt-like phenotype with increased levels of phosphorylated NCC, thus suggesting that these short TSC22D isoforms may exert a negative effect on NCC (*28*). This motivated us to investigate the effect of TSC22D3 on the WNK4-SPAK pathway. We observed that the expression of TSC22D3.1 decreased the phosphorylation of SPAK in cells expressing SPAK, WNK4, and NRBP1 (Fig. 9, A and B). A similar inhibitory effect of TSC22D3.1 was observed in the presence of overexpressed TSC22D2.

To assess the differential and/or possibly opposing effect of a short versus a long TSC22D protein on WNK condensate formation, we expressed TSC22D2 (a long TSC22D isoform) and TSC22D3.1 (a short TSC22D isoform) alone or in combination, together with WNK4 and SPAK, and observed their effects. When cotransfected with WNK4 alone, SPAK was localized in cytoplasmic condensates (Fig. 9C). SPAK localization was similar when TSC22D3 was added, while TSC22D3 showed a diffuse cytoplasmic localization (i.e., not in WNK condensates) (Fig. 9D). In contrast, overexpression of TSC22D2 with SPAK and WNK4 (in the absence of TSC22D3) resulted in condensates in which SPAK and TSC22D2 colocalized. These were larger and more irregular in shape, supporting a pro-condensation role, or a condensate modifying property for long TSC22D proteins (Fig. 9E). Last, in the presence of all components (WNK4, TSC22D3, and TSC22D2), SPAK localization was diffuse in most cells (Fig. 9F). However, condensates were still present in some cells, and these condensates were positive for both TSC22D2 and TSC22D3 (not shown).

**Fig. 10.**
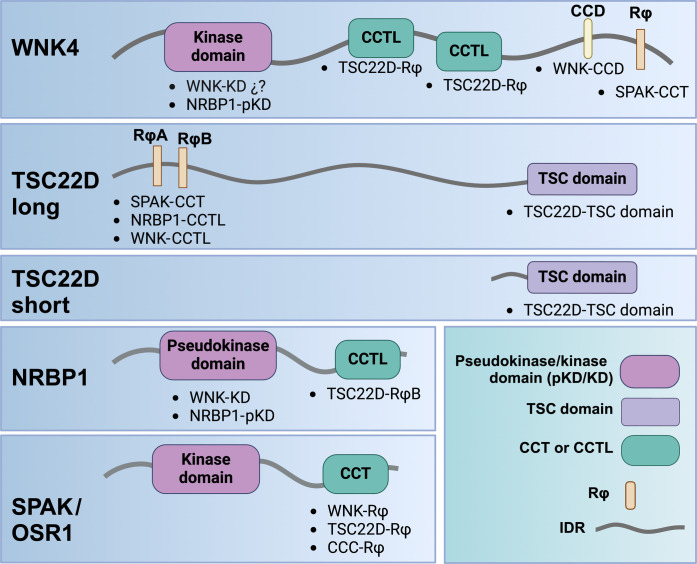
Motifs and domains mediating interactions between components of the WNK-SPAK/OSR1-TSC22D-NRBP pathway. Below the indicated domains and motifs, we list the specific domains or motifs with which there is experimental validation of interaction or prediction of interaction from structural models. WNKs contain one or more Rϕ motifs within their C-terminal domain that interact with the CCT domain of SPAK (*16*, *17*, *33*). WNKs also contain two CCTL domains in their C terminus that mediate interaction with Rϕ motifs in long TSC22D proteins [this work and (*12*)]. The absence of this interaction may explain the loss of function effect of mutations within these domains (*16*, *47*). Last, WNKs can homo- and heterodimerize via their C-terminal coiled-coil domain (*46*) or through kinase domain–mediated interactions (*48*). WNKs can also interact with NRBP1 apparently via a kinase-pseudokinase domain mediated interaction (*12*). SPAK can also interact with CCCs through a SPAK CCT-domain–CCC-Rϕ interaction (*49*, *50*) and with TSC22Ds through a SPAK-CCT-domain–TSC22D-Rϕ interaction (*15*). We speculate that the presence of TSC22D2 (Fig. 1) and NRBP1 (Fig. 5) in the apical membrane of DCT cells may be due to this SPAK-TSC22D interaction. Long TSC22D proteins can also interact with the CCT domain of NRBP1 via an Rϕ motif (*11*–*13*). Last, long and short TSC22D proteins can homo- and heterodimerize via their C-terminal TSC domain (*12*). We speculate that the inhibitory effect of TSC22D3 on the WNK-SPAK/OSR1 pathway (Fig. 9) (*28*) may be due to its heterodimerization with long TSC22D proteins, thus somehow preventing their activating effect. Created in BioRender: M. Castaneda bueno (2024), https://BioRender.com/c08f700.

We also observed that TSC22D3 can interact with TSC22D2 in immunoprecipitation assays (Fig. 9G). This interaction is most likely mediated by the TSC22D heterodimerization domain as suggested by data from our parallel work (Fig. 10) (*12*), and, thus, we hypothesize that prevention of TSC22D2 dimerization in the presence of TSC22D3 may explain the inhibitory effect of the latter. This hypothesis was supported by the observation that stimulation of TSC22D2 self-oligomerization prevented the inhibitory effect of TSC22D3. Self-oligomerization of TSC22D2 was achieved by fusing TSC22D2 to Cry2-Clust, a light-responsive oligomerization domain from *Arabidopsis Thaliana* (Fig. 9H) (*36*).

In summary, our observations suggest that short TSC22D isoforms may oppose the effects of long TSC22D isoforms on the WNK-SPAK/OSR1 pathway. In our system, TSC22D3 appeared to oppose the pro-condensation effect of TSC22D2 (perhaps through heterodimerization), and this effect may be related to its negative effect on pathway activation.

## DISCUSSION

The very recent identification by us and an independent group of the functional interaction of NRBP1 and TSC22D proteins with the WNK-SPAK-CCC pathway (*11*, *12*) motivated our study of the role of these proteins in the regulation of WNK signaling, NCC activity, and DCT physiology. First, we showed that TSC22D2 and TSC22D1.1 expression is enriched in the DCT when compared to other nephron segments (Fig. 1), consistent with previous transcriptomic and proteomic studies. This further highlights the relevance of this signaling pathway in these cells where WNK4 and KS-WNK1 are also enriched (*26*, *27*). Moreover, our data showed that, under different conditions in which WNK bodies are formed in the DCT and NCC is activated (e.g., LKD), TSC22D1.1, TSC22D2, and NRBP1 localize within WNK bodies. This constitutes an additional piece of evidence, suggesting the similarity of the low K^+^-induced DCT WNK bodies and the WNK condensates that are induced by hypertonicity in different cell types as part of the cell-volume regulatory response (*11*).

Second, our data suggest that NRBP1 and TSC22D proteins can exert an activating effect on the WNK4-SPAK/OSR1 pathway by promoting an increase in the autophosphorylation of WNK4 (Fig. 4). This is consistent with the observation by Xiao *et al.* (*11*) that, like WNK1, TSC22D2 and NRBP1 also participate in cell volume regulation and are functionally related as revealed by coessentiality analysis. It is also consistent with the observation that *NRBP1* KO in cultured cells reduces pSPAK and phosphorylation of WNK1 at the activating Ser^382^ phosphorylation site and that, in in vitro kinase assays performed with recombinant WNK4 (1-449), NRBP1 presence increased the T-loop autophosphorylation of WNK4 as well as the WNK4-mediated OSR1 phosphorylation (*12*). This latter result, however, is slightly different from our observations made in the HEK293 assay because, in these cells, we only observed WNK4 activation in the presence of both NRBP1 and long TSC22D proteins, but not in the presence of NRBP1 alone (Fig. 4). This was particularly evident in the dose-response experiments, in which the addition of increasing amounts of NRBP1 did not increase pSPAK levels, but addition of NRBP1 together with TSC22D2 had a robust activating effect (fig. S4). The contrast in these observations is likely due to the different nature of the assays (e.g., in vitro context versus cellular context). Our studies indicate that TSC22D isoforms are likely to be required for NRBP1 to maximally activate WNK4 and likely other WNK isoforms. In future work, it would be important for the impact of TSC22D isoforms to be assessed in the in vitro activation studies.

We speculate that a direct interaction between the WNK kinase domain and the NRBP1 pseudokinase domain may occur and that, like for other pseudokinases that have regulatory activity on their related kinases, the pseudokinase (NRBP1) may adopt an active conformation in response to a stimulus (e.g., hypertonicity) that permits its interaction with the kinase (WNKs) leading to its allosteric activation. This proposal is based on interaction models presented in a parallel study (*12*), but this hypothesis remains to be tested experimentally. The requirement for both a long TSC22D protein and NRBP1 to observe activation in the cellular HEK293 assay suggests that TSC22D interaction with NRBP1 may be required to achieve the conformation necessary for interaction with WNK in vivo. Another possibility is that, given that TSC22D proteins are highly disordered and appear to contribute to condensate formation (Fig. 7), overexpression of both NRBP1 and long TSC22Ds promote the WNK-NRBP1 interaction to occur within biomolecular condensates where the high concentrations of components of this pathway may enhance WNK kinase activation. Alternatively, WNK condensates may constitute an environment with specific physicochemical properties that may favor WNK activation by NRBP1.

The modulatory role of NRBP1 and TSC22D proteins on the WNK-SPAK/OSR1 pathway was demonstrated in vivo, as the inducible deletion of NRBP1 specifically in the DCT reduced the phosphorylation of NCC (Fig. 5). NRBP2 presence in these cells likely exerts some compensatory effect, as our data show that NRBP2 can also promote WNK-SPAK/OSR1 activation (fig. S3), and NRBP2 presence was clearly observed in DCT cells (Fig. 5). Moreover, increased WNK bodies’ size in the KO mice was probably also part of a compensatory response. Increased presence of WNK bodies has been observed in other models with reduced NCC activity like *Wnk4* KO mice and *Cab39*/*Cab39L* double-KO mice (*25*, *37*). In the present study, increased TSC22D1.1 kidney abundance was observed in *WNK4* KO mice, which might also be part of the compensatory response that occurs in these mice.

Previous works have shown other functional roles of NRBP1 and TSC22D proteins. For example, NRBP1 has been proposed to function as a substrate adaptor molecule for Cullin-Ring ligase (CRL) complexes with ubiquitin ligase activity (*21*, *38*). Whether this function is related to their ability to modulate the WNK-SPAK/OSR1 signaling pathway remains to be determined. However, the observation that NRBP1 binding to CRL elements (e.g., Elongins B and C) is not modulated by hypertonicity like interaction with WNKs (*12*) suggests that these may constitute independent functions. One of the proposed targets for a NRBP1-containig CRL complex is SALL4, a transcription factor that is key in mediating stem cell fate (*38*, *39*). Inducible KO of NRBP1 in intestinal progenitor-like cells of mice caused aberrant proliferation of these cells and SALL4 was found to be increased (*38*). A SALL4 paralog, SALL3 is highly enriched in the DCT (*26*, *40*). Our data show that SALL3 can bind NRBP1 in vitro (fig. S11), but we did not observe changes in SALL3 expression or localization in the DCTs of NRBP1 KO mice (fig. S11). Further work is necessary to explore the possible role of NRBP1 in SALL3 regulation in the DCT. In addition, the evidence of the role of NRBP1/TSC22Ds in the regulation of cell proliferation is extensive. For instance, global KO of *Nrbp1* in mice was observed to produce an increased incidence of tumorigenesis (*38*) and decreased NRBP1 expression has been observed in a wide variety of tumors (*38*, *41*). In *Drosophila*, the NRBP1 homolog Mlf-1-adapter-molecule (MADM) and the long TSC22-like protein Bunched A have been shown to promote growth (cell number and cell size) (*13*, *42*). Whether these roles are related to WNK modulation, CRL function, or both remains uncertain. Further exploration of the DCT-specific KO mice will be necessary to assess whether NRBP1 absence affects DCT cell proliferation.

Last, we also investigated the role of specific domains and motifs involved in the interactions among the components of this pathway. Mainly, we focused on defining the role of each interaction domain/motif present in WNK4. The presence of a single validated Rϕ motif in WNK4 simplified this analysis. Our data are consistent with the notion that the CCT domain–Rϕ motif interaction mechanism comprises the dominant mechanism mediating site-specific interactions among components of this pathway. A summary of the current knowledge is presented in Fig. 10. An AlphaFold model has been generated, in which most of these experimentally validated interactions are predicted (*12*). It remains to be determined whether this structured complex can exist within WNK biomolecular condensates. Our data provide at least some evidence that there is certain flexibility regarding the specific interactions that can be used to recruit pathway components to the condensates and promote pathway activation. For instance, it was observed that, in the absence of direct WNK-TSC22D interaction (WNK4-CCTL1,2 mutant), the SPAK-WNK4 interaction could recruit WNK4 into TSC22D2 containing condensates, and, thus, TSC22D/NRBP1-mediated activation of WNK4 could occur (Figs. 6 and 8). Furthermore, fusion of the exogenous interaction modules FRB and FKBP12 to TSC22D2 and to the WNK4 triple mutant that is incapable of binding SPAK or TSC22Ds, respectively, rescued the interaction in the presence of rapamycin and promoted pathway activation (Fig. 8, F to I).

## MATERIALS AND METHODS

### Mice studies

Animal studies were approved by the Animal Care and Use Committee of the Instituto Nacional de Ciencias Médicas y Nutrición Salvador Zubirán (approval number NMM-2094-23-29-1). For immunolocalization studies, male and female WT mice were given LKD (0% K^+^; Research Diets, D16120202Mi) or NKD (0.8% K^+^; that was prepared by adding KCl to the LKD) for 7 days. *Klhl3*-R528H homozygous mice (*24*) and *Wnk4* KO homozygous mice (*7*) were also studied under baseline conditions (normal chow).

DCT-specific, *Nrbp1*-inducible KO mice were generated as follows. Sperm from the C57BL/6 N-A<tm1Brd>Nrbp1<tm3a(EUCOMM)Wtsi>/WtsiOulu strain was obtained from the EMMA repository (EMMA strain ID: 09610). In vitro fertilization was performed to rederive the strain (fertilization and implantation of C57BL6/J oocytes into pseudopregnant C57BL/6J females). Heterozygous progeny carrying the 3-loxP *Nrbp1* allele (identified by polymerase chain reaction of tail genomic DNA) were then crossed with Flippase-enhanced (FLP-e) knock-in mice [Jackson Laboratory, stock no. 016226; B6N.129S4-Gt(ROSA)^26Sortm1(FLP1)Dym^/J] to eliminate the neomycin cassette, the third loxP site, and generate the conditional-ready allele (TM3c, www.eummcr.info/faq#nomenclature_eucomm). After confirming FlpE-mediated recombination, mice harboring the conditional-ready allele were bred with NCC-Cre mice (*31*). For experiments, homozygous NRBP1 floxed mice that were also heterozygous for the NCC-Cre allele (*Nrbp1^fl/fl^;NCC^CreERT/−^*) were treated with five doses of tamoxifen (100 mg/kg per day) given every other day to induce the deletion (*31*). WT controls were *Nrbp1^wt/wt^;NCC^CreERT/−^*, *Nrbp1^wt/fl^;NCC^CreERT/−^*, or *Nrbp1^fl/fl^;NCC*^*CreERT*/−^ that were treated with vehicle [100 μl/20 g of body weight of sterile corn oil (Thermo Fisher Scientific, no. AC405435000)]. After tamoxifen treatment, LKD (0% K^+^) was given to the mice for 5 days, after which mice were euthanized.

Tissue collection was performed in the following way. Animals were anesthetized with isoflurane (2%). The left kidney was harvested for immunoblot studies. The right kidney was perfused with 20 ml of phosphate-buffered saline (PBS) followed by 20 ml of 4% (w/v) paraformaldehyde (PFA) in PBS for immunofluorescence studies. Harvested kidneys were incubated for 3 hours in 4% PFA and then overnight in 30% (w/v) sucrose in PBS at 4°C. Tissues were mounted in optimum cutting temperature (OCT) reagent (Tissue-Tek), and 5-μm sections were cut and stored at −80°C.

### Immunofluorescent staining of kidney tissue

For immunostaining, tissue sections were washed with tris-buffered saline (TBS) containing 0.1% Tween 20 (TBSt), followed by blocking with 10% (w/v) bovine serum albumin (BSA) in TBSt for 30 min at room temperature. Sections were incubated overnight at 4°C with primary antibodies diluted in TBSt containing 10% BSA, followed by secondary antibodies for 1 hour at room temperature. After washes, sections were mounted using VECTASHIELD Vibrance mounting medium (Vector Laboratories) to preserve fluorescence. Fluorescent images were acquired using a Leica DMi8 confocal microscope or an Echo Revolve R4 epifluorescence microscope.

Image processing was performed using ImageJ. For image analysis, *Z*-stacks of immunostained kidney sections from WT or *Nrbp1* KO animals (*n* = 3 per group, both sexes) were combined and converted into maximum intensity *Z*-projections. Processed images were thresholded, despeckled, and analyzed by manually selecting regions of interest (ROIs) for puncta quantification and morphological measurements. Circularity was calculated using the formula: Circularity = 4π × Area/(Perimeter^2^), where values closer to 1.0 indicate a more circular shape. To quantify apical pSPAK and SPAK signal, ROIs encompassing the apical membrane of NCC-positive tubules were selected, and mean fluorescence intensity was measured.

### Western blotting of kidney protein samples

Kidneys were homogenized with lysis buffer containing 250 mM sucrose, 10 mM triethanolamine, 50 mM sodium fluoride, 5 mM sodium pyrophosphate, 1 mM sodium orthovanadate, 10 mM 1,10-phenanthroline, and cOmplete protease inhibitor cocktail (Roche). Protein concentration was quantified by the bicinchoninic acid (BCA) protein assay (Pierce). Proteins were prepared in 1× Laemmli buffer and loaded into SDS–polyacrylamide gel electrophoresis (PAGE), transferred to polyvinylidene difluoride membranes, and blocked for 1 hour with 10% (w/v) nonfat milk in TBSt. Antibodies were diluted in 5% (w/v) nonfat milk in TBSt. Incubation with primary and horseradish peroxidase–coupled secondary antibodies was performed overnight at 4°C and 1 hour at room temperature, respectively. Enhanced chemiluminescence reagent was used for signal detection. For band analysis, raw images from the immunoblots were subjected to a densitometric analysis in ImageJ. Values were then normalized to the control samples, and relative values were calculated for each experimental group.

### Cell experiments and immunoblots

HEK293 [American Type Culture Collection (ATCC), CRL-1573] cells were transiently transfected with expression plasmids (see table S2). Cells were grown at 37°C, with 5% CO_2_, in Dulbecco’s modified Eagle’s medium (Gibco) with 10% fetal bovine serum, to a 70 to 80% confluence and then transfected with Lipofectamine 2000 (Life Technologies). All experiments were carried out in isotonic conditions unless otherwise stated. Forty-eight hours after transfection, cells were lysed with a lysis buffer containing 50 mM tris-HCl (pH 7.5), 1 mM EGTA, 1 mM EDTA, 50 mM sodium fluoride, 5 mM sodium pyrophosphate, 1 mM sodium orthovanadate, 1% (w/v) Nonidet P-40, 270 mM sucrose, and protease inhibitors (cOmplete tablets; Roche Applied Science and 10 mM 1,10-phenanthroline). Protein concentration was quantified using BCA (Bio-Rad) assay, and Western blots were performed as described above.

For certain experiments, cells were stimulated before lysis as indicated in the figure legends. Briefly, stimulation with rapamycin (1 μM) was performed for 30 min. Stimulation with blue light for experiments with CRY2-TSC22D2 was performed for 30 min in a blue light light-emitting diode chamber (460 to 490 nm). All cell groups from these experiments were seeded in a multi-well plate and were stimulated simultaneously.

For live microscopy studies, cells were seeded in glass bottom culture dishes (Nest, 801002), and transfection was performed as explained above. Most of these experiments were performed in COS7 cells as condensates were more readily detectable in this cell line, although some experiments were also performed in HEK293 cells. Cell line used for each experiment is indicated in the figure legend from each experiment. Constructs with fused fluorescent tags were used. Pictures of cells were taken 24 to 48 hours posttransfection. For some experiments, simultaneous observation of multiple transfected proteins was achieved through observation of fluorescent label-tagged proteins and immunostaining of other proteins as indicated in the figure legends for each experiment. For immunostaining in cells, 24 hours after transient transfection, cells were treated with PBS^++^ (containing 0.9 mM CaCl_2_ and 0.5 mM MgCl_2_) and subsequently fixed with 2% PFA in PBS^++^ for 15 min. Following fixation, cells were washed twice with PBS^++^. Next, they were incubated with a blocking solution (PBS^++^ containing 10% w/v BSA and 0.3% Triton X-100) for 1 hour at room temperature. The primary antibody was diluted in the blocking solution and incubated with the cells at 4°C overnight. After three washes with PBS^++^ containing 0.2% Tween 20, cells were incubated in the dark with mouse anti-rabbit DyLight 405-nm antibody (diluted 1:150 in blocking solution) for 1 hour at room temperature. Following three additional washes with PBS^++^ containing 0.2% Tween 20, mounting medium (VECTASHIELD Vibrance in PBS^++^, 1:20) was added to the cells.

Live-cell imaging of transfected COS7 cells stimulated with hypotonic and hypertonic medium was performed as follows. Transfected cells were visualized 48 hours after transfection using an Echo Revolve R4 microscope. Cells were recorded before, during, and after addition of water to the medium to induce hypotonic stress (200 mosmol). For stimulation with hypertonic stress, cells transfected with WNK4-mCherry and SPAK-GFP in which no condensates were observed at baseline were identified. The formation of condensates was observed after addition of sorbitol (final concentration, 250 mM).

### Immunoprecipitation and coimmunoprecipitation experiments

Immunoprecipitation of recombinant FLAG-tagged proteins was performed with FLAG M2 Magnetic Beads (Sigma-Aldrich) following the manufacturer’s instructions. Briefly, 1 mg of protein extract was prepared and diluted with immunoprecipitation buffer [1× TBS, 1% Triton X-100, 1 mM EDTA (pH 7.5), 1 mM sodium orthovanadate, 10 mM sodium pyrophosphate, and 50 mM sodium fluoride]. Magnetic FLAG beads (30 μl) were added and incubated at 4°C for 2 hours, after which they were washed three times with 500 μl of 1× TBS buffer with 0.05% Tween 20. Bound proteins were then eluted with a denaturing buffer containing SDS, glycerol, bromophenol blue, and β-mercaptoethanol (2× Laemmli).

Immunoprecipitation of recombinant hemagglutinin (HA)–tagged proteins was performed with HA Magnetic Beads (Pierce) following the manufacturer’s instructions. Briefly, 15 μl of anti-HA magnetic beads were washed three times with wash buffer (TBS and 0.1% Tween 20), and then 1 mg of lysate was added. The suspension was incubated at room temperature for 20 min. After incubation the beads were washed three times then eluted in Laemmli buffer. For coimmunoprecipitation experiments, eluted proteins were subjected to SDS-PAGE and immunoprecipitated, and coimmunoprecipitated proteins were detected by immunoblots using tag-directed antibodies as indicated.

### Generation of SLC12 KO HEK293 cells

NKCC1 and KCCs were sequentially targeted for inactivation in HEK293 (ATCC, CRL-1573) cells. In a first step, guide CCGCTTCCGCGTGAACTTCG targeting *SLC12A2* at exon 1 was inserted into vector pX458 at the Bbs I site. HEK293 cells were then transfected with this targeting vector. The pX458 vector expresses the guide RNA under a U6 promoter and enhanced green fluorescent protein (EGFP) fused to cas9 under a chicken β-actin promoter. Two days posttransfection, cells were fluorescence-activated cell sorting (FACS) sorted using EGFP fluorescence as a marker of vector expression, and single green cells were plated in 2× to 3× 96-well plates. Clones were allowed to grow, transferred to 24-well plates, and tested for NKCC1 function. One clone lacking bumetanide-sensitive K^+^ influx (fig. S9A) was selected and used for targeting K-Cl cotransport function. Note that, under regular conditions, there is no KCC function detectable in HEK293 cells. The function can be elicited by exposing cells to the WNK inhibitor WNK463 (fig. S9B, bars 1 to 3). Because transcript abundance of all four K-Cl cotransporters is similar in HEK293 cells [Human Protein Atlas; (*43*)], we targeted the SLC12A4-7 genes at once. The following guides were selected: SLC12A4 (GGGCAGGTACACCCCCATGA), SLC12A5 (CGGCAGGTACACGCCCATGA), SLC12A6 (TGGGAGGTAGACACCC ATGA), and SLC12A7 (CGGCAGGTAGACGCCGATGA). Cells were transfected with equal amounts of each pX458 vector and FACS sorted 2 days later, and clones were isolated and tested for function. Clone HEK293-ΔKCC-26 was selected and further characterized. As seen in fig. S9B (bars 5 and 6), no activation of K^+^ influx is observed in these cells with WNK463, demonstrating the absence of K-Cl cotransporter function.

### ^83^Rb uptake experiments

K^+^ influx measurements were performed using ^83^Rb as a tracer (Brookhaven National Laboratories, Upton, NY). Before the experiment, cells were plated in 24-well plates pre-coated with poly-l-lysine (0.1 mg/ml). They were then exposed to isosmotic or hyperosmotic (NKCC1 function) saline for 15 min of preincubation in the presence or absence of 1 μM WNK463 (Sigma-Aldrich). The isosmotic saline contained 140 mM NaCl, 5 mM KCl, 1 mM CaCl_2_, 0.8 mM MgSO_4_, 1 mM glucose, and 10 mM Hepes (pH 7.4). The hyperosmotic saline was of identical composition but contained 80 mM sucrose. After aspiration, the preincubation solution was replaced with identical saline containing 100 μM ouabain, ^83^Rb (0.25 μCi/ml) with or without 20 μΜ bumetanide (NKCC1 function), 1 μM WNK463 (to activate KCC function), and 10 μM ML077 (VU0240551, to inhibit KCC function). Before the flux experiment started, two 5-μl aliquots of radioactive saline were sampled, added to vials containing 5 ml of scintillation fluid, and used as standards. Each flux condition was measured in triplicate. The uptake was terminated after 15 min by aspiration of the radioactive solution and three rapid washes with ice cold saline. Cells were then lysed with 500 μl of NaOH (0.2 M) for 1 hour and then neutralized with 250 μl of acetic acid glacial. Lysates (150 μl for scintillation counting and 20 to 30 μl for protein assays) were sampled. The protein assay used was Bio-Rad Bradford reagent according to the manufacturer’s instructions. K^+^ influx was calculated and expressed in nanomole of K^+^ per milligram of protein per min.

### Generation of a long-WNK1 (L-WNK1) KO HEK293 cell line

The PX458 polycistronic plasmid was used that allows for simultaneous SpCas9, GFP, and guide RNA (gRNA) expression as described in the protocol by Ran *et al.* (*44*). Briefly, a single guide RNA targeting exon 1 of WNK1 (sequence TCCAGCGAACCGACCATGTC), selected with the Chopchop web tool for gRNA specificity and efficiency maximization (https://academic.oup.com/nar/article/47/W1/W171/5491735), was cloned into the PX458 plasmid. This was confirmed by Sanger sequencing. HEK293 cells were transfected with this plasmid as described in a previous section, detached 48 hours later, and subjected to FACS based on GFP fluorescence for single-cell isolation into a 96-well plate. After clonal expansion, cells were subjected to Western blot to assess the levels of WNK1 abundance and kinase activity by the measurement of pSPAK-S371 phosphorylation (the S motif site, previously identified as S373; see Q9UEW8 in UniProt). The C3 clone was selected as the KO cell line, as undetectable levels of WNK1 were found, as well as clearly reduced levels of SPAK phosphorylation (fig. S12).

### Plasmids

All modified constructs (e.g., WNK4 mutants, FKBP12 and FRB fusion proteins, and tagged proteins) were generated by Fast Cloning (*45*). Fast cloning was performed using the Phusion-Plus high-fidelity DNA polymerase and confirmed by Sanger sequencing or whole-plasmid sequencing (Plasmidsaurus, USA). For plasmid information see table S2. pSpCas9(BB)-2A-GFP (PX458) was a gift from F. Zhang (Addgene, plasmid no. 48138; http://n2t.net/addgene:48138; RRID:Addgene_48138)

### Antibodies

Antibody sources, dilutions, and validation data and references are provided in table S3 and fig. S13. Additional validation performed with KO cell lines for some of the TSC22Ds antibodies and the NRBP1 antibody is presented in (*12*).

### Statistical analysis

All statistical analyses were performed using R (version 2024.04.2+764). For comparisons between two independent groups, Student’s *t* test was applied to evaluate statistical significance. Data are presented as means ± SD or as box plots representing the median and interquartile range. Statistical significance was defined as *P* < 0.05 for all tests. For comparisons among multiple groups, one-way analysis of variance (ANOVA) tests were conducted followed by Tukey’s post hoc tests. For data that do not follow a normal distribution (condensate size and circularity), Wilcoxon tests were performed. Scheirer-Ray-Hare tests followed by pairwise Wilcoxon post hoc test were performed to evaluate interaction between genotype and sex.

## Supplementary Materials

### The PDF file includes:

Figs. S1 to S13 Tables S1 to S3 Legends for movies S1 and S2 Legends for data S1 to S10 References
