# Supplementary material for: NRBP1 and TSC22D proteins affect distal convoluted tubule physiology through modulation of the WNK pathway
Source: Sci Adv. 2025 Jul 16;11(29):eadv2083. doi: 10.1126/sciadv.adv2083 (PMC12266109; doi:10.1126/sciadv.adv2083)
Supplement: Supplementary file 1 — Figs. S1 to S13 Tables S1 to S3 Legends for movies S1 and S2 Legends for data S1 to S10 References [file sciadv.adv2083_sm.pdf]

Science Advances
AAAS

Supplementary Materials for

NRBP1 and TSC22D proteins affect distal convoluted tubule physiology through modulation of the WNK pathway

Germán Magaña-Ávila *et al.*

Corresponding author: María Castañeda-Bueno, maria.castanedab@incmnsz.mx, mcasta85@yahoo.com.mx

Sci. Adv. **11**, eadv2083 (2025)
DOI: 10.1126/sciadv.adv2083

The PDF file includes:

Figs. S1 to S13
Tables S1 to S3
Legends for movies S1 and S2
Legends for data S1 to S10
References

Other Supplementary Material for this manuscript includes the following:

Movies S1 and S2
Data S1 to S10

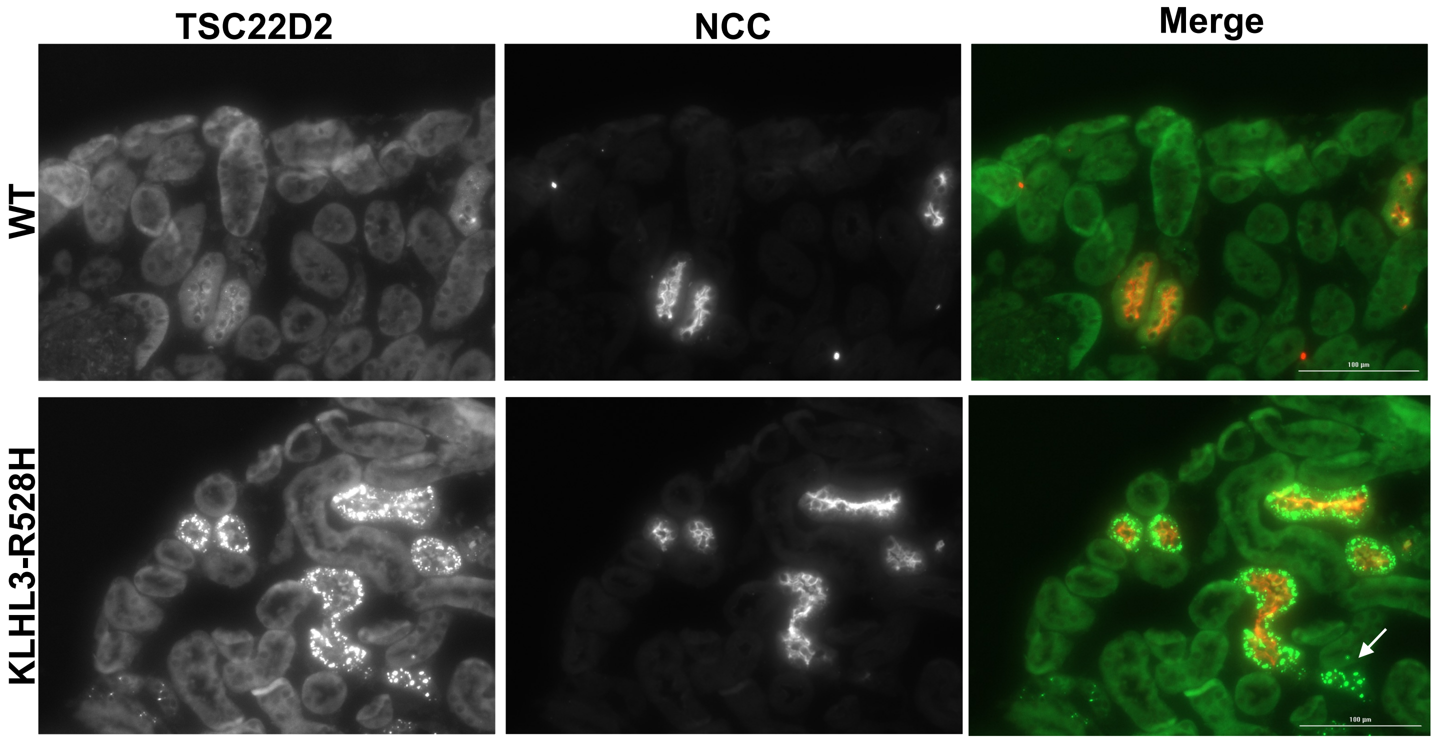

Fig. S1. TSC22D2-positive condensates are also observed in sporadic NCC-negative cells. Kidney tissue slices from wild type and KLHL3-R585H homozygous knockin mice were co-stained with antibodies against NCC and TSC22D2. With the TSC22D2 antibody, condensates were mainly observed in the cytoplasm of DCT cells from KLHL3-R528H knockin mice, but also in a few NCC-negative tubules. The white arrow indicates an example of such a tubule.

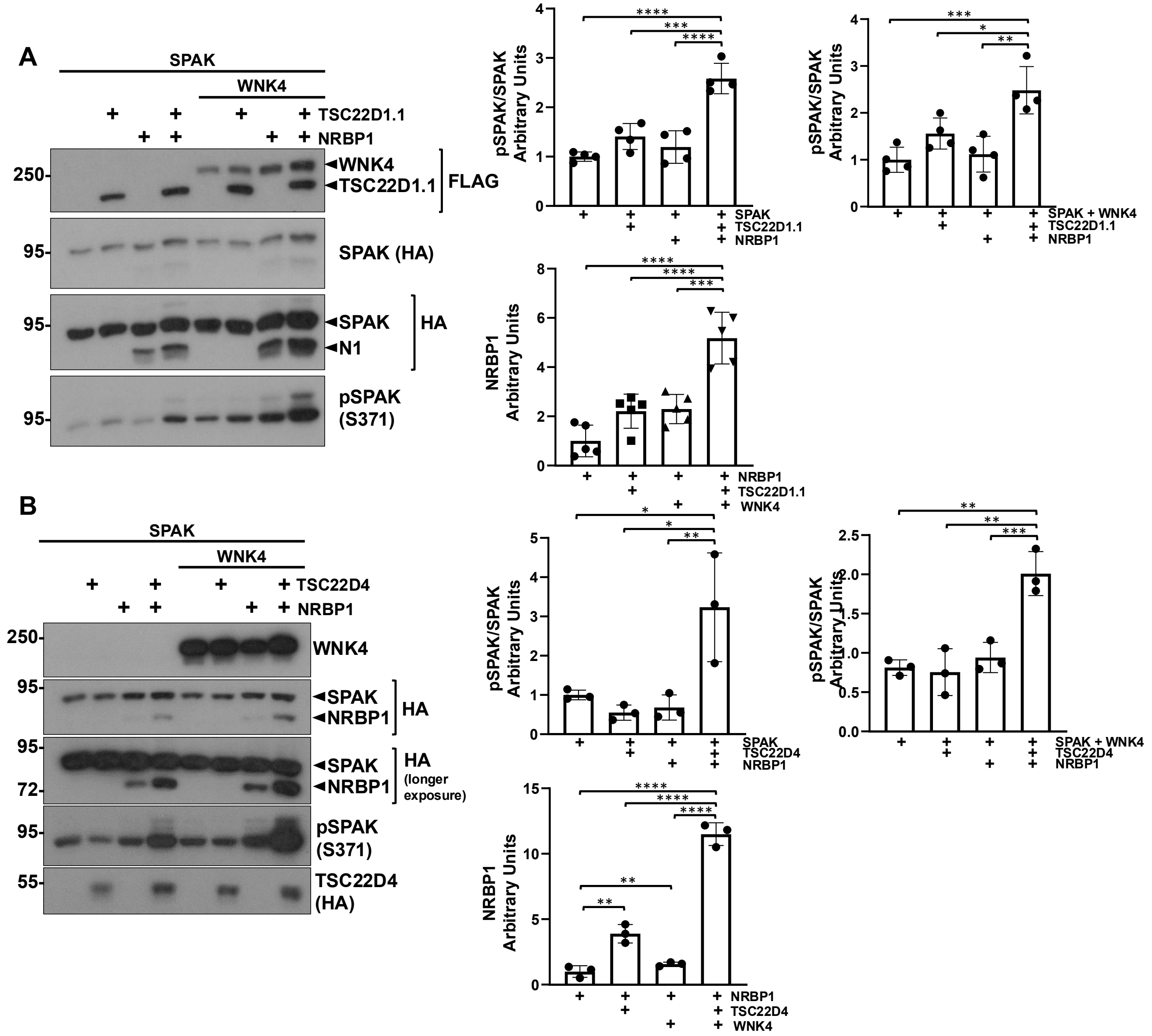

Fig. S2. NRBP1 acts in concert with long TSC22D isoforms to promote WNK-mediated SPAK phosphorylation. The effect of co-expression of NRBP1 with TSC22D1.1 **(A)** or TSC22D4 **(B)** on endogenous WNK's and WNK4´s ability to phosphorylate SPAK was assessed in HEK293 cells. Cells were transiently transfected with SPAK, WNK4, NRBP1 and the different TSC22D long isoforms as indicated. 48 hours post transfection immunoblots were performed to confirm expression of transfected proteins and to analyze phosphorylation levels of SPAK. An increase in SPAK phosphorylation levels was observed upon co-expression of NRBP1 and TSC22D1.1 or TSC22D4 in the absence of overexpressed WNK, perhaps due to an effect on the endogenous WNK. In the presence of WNK4, SPAK phosphorylation increased upon co-expression of NRBP1 with both long TSC22D proteins. Results of quantitation are shown in the graphs to the right. ANOVA followed by Tukey post hoc tests were performed to identify statistically significant differences. *p<0.05, **p<0.01, ***p<0.001, ****p<0.0001. At least three independent experiments were performed with each TSC22D isoform.

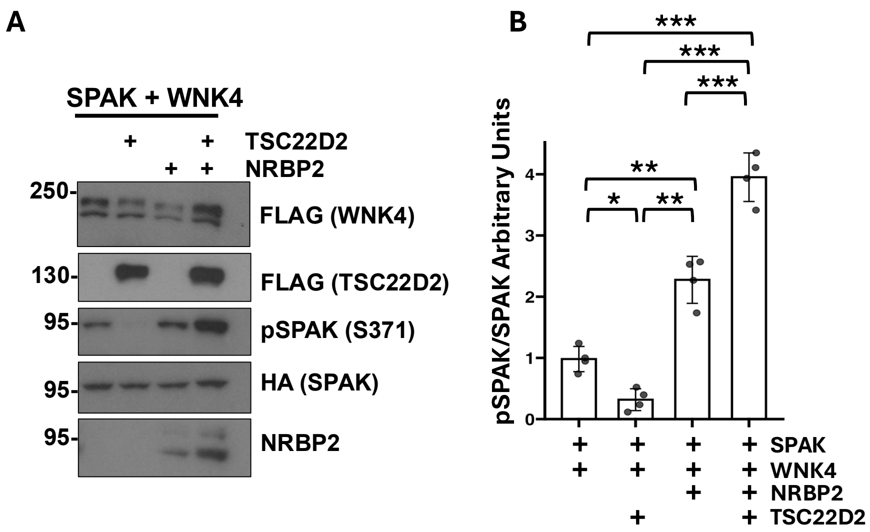

Fig. S3. NRBP2 also promotes WNK-SPAK pathway activation. **(A)** The effect of NRBP2 on WNK4-mediated SPAK phosphorylation was assessed in the absence and presence of TSC22D2. Cells were transiently transfected with SPAK, WNK4, NRBP2 and TSC22D2, as indicated. Co-expression of NRBP2 with TSC22D2 promoted an increase in the levels of pSPAK. **(B)** Results of quantitation of blots represented in **(A)**. ANOVA followed by Tukey post hoc tests were performed to identify statistically significant differences. *$p<0.05$, **$p<0.01$, ***$p<0.001$. At least three independent experiments were performed.

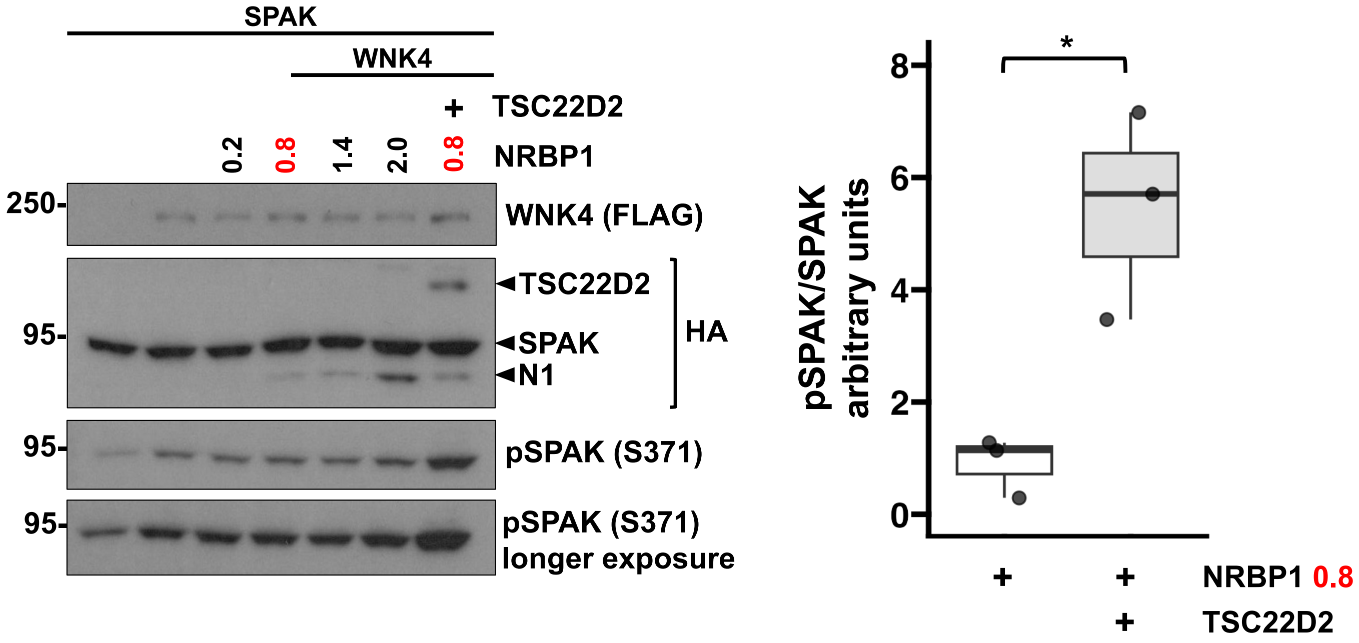

Fig. S4. Comparison of the effect on SPAK phosphorylation of increased abundance of NRBP1 vs. co-expression of NRBP1 and TSC22D2. HEK293 cells were transfected with SPAK, WNK4, and increasing amounts of NRBP1 as indicated. In addition, one group of cells was transfected with WNK4, SPAK, 0.8 µg of NRBP1 DNA, and TSC22D2. Despite observing a higher abundance of NRBP1 in the group transfected with the highest amount of NRBP1 alone than in the group with NRBP1 + TSC22D2, a clearly greater pSPAK signal was observed in the latter group. This suggests that the additive effect on SPAK phosphorylation of NRBP1 and TSC22D2 co-expression was not due to the effect of TSC22D2 on NRBP1 abundance. At least three independent experiments were performed with similar results. Student t test was performed to compare groups transfected with 0.8 µg of NRBP1 expression plasmid; *$p<0.05$.

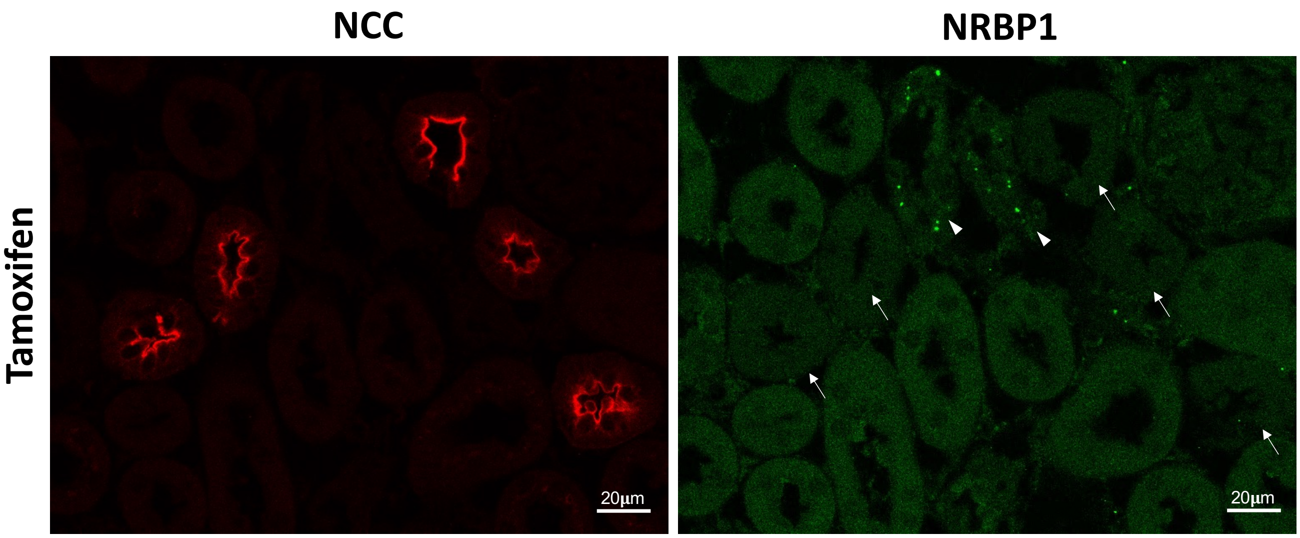

Fig. S5. NRBP1 positive tubules in DCT-specific knockout mice. Immunofluorescent staining of kidney sections from DCT-specific NRBP1 knockout mice. NRBP1-positive condensates were observed in sporadic cells that were NCC-negative (arrowheads), supporting the specificity of the cell type-specific targeting strategy and confirming that NRBP1-positive condensates are present in certain non-DCT cells. Also noticeable in this image is that NCC positive tubules (arrows) have no NRBP1-positive condensates and have a less intense diffuse cytoplasmic staining than surrounding tubules.

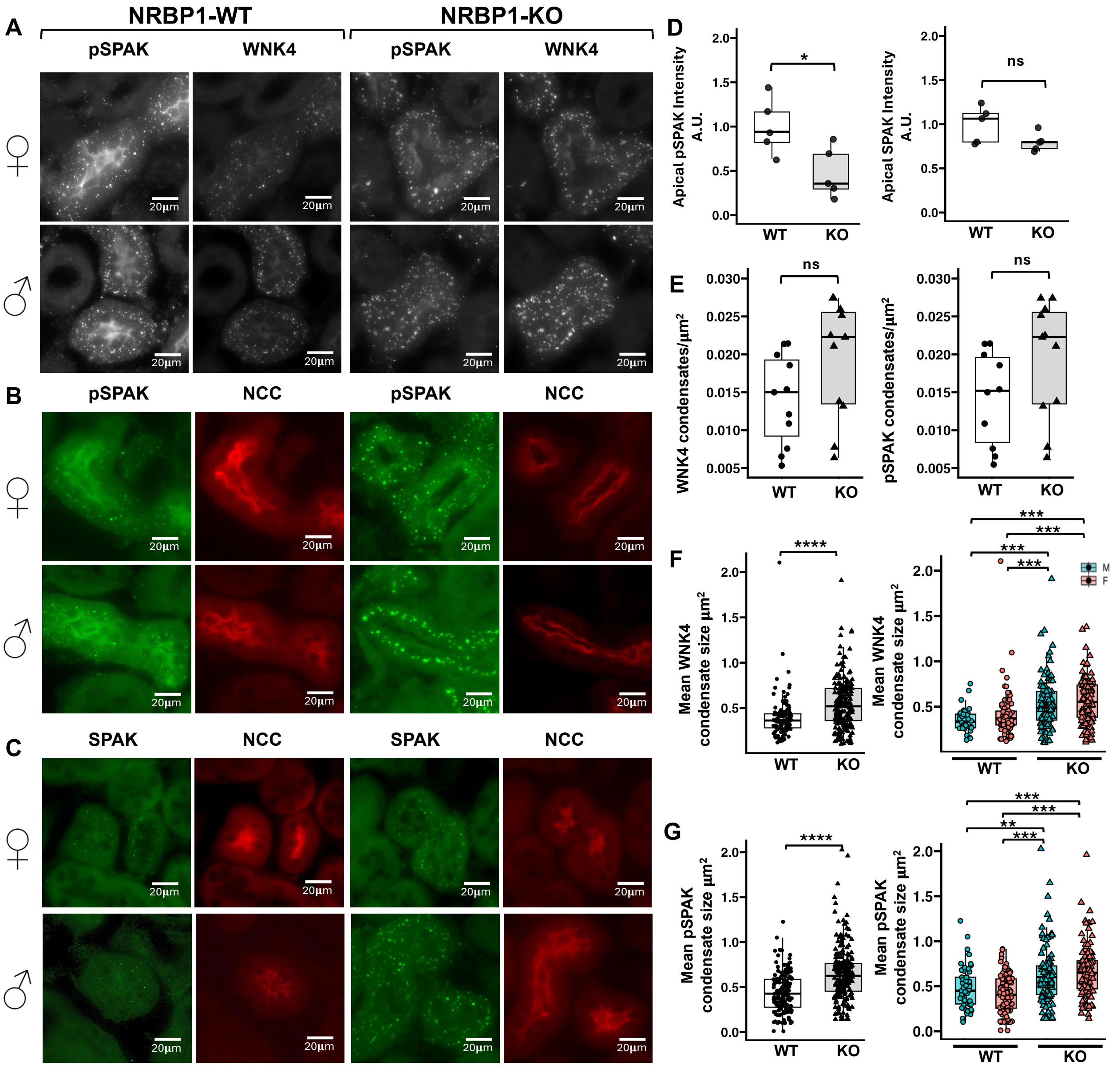

Fig. S6. DCT-specific NRBP1 knockout mice have reduced phosphorylated SPAK in the apical membrane. Immunofluorescent staining of kidney sections from DCT-specific NRBP1 knockout mice was performed. **(A and B)** Under low K^+ diet, less pSPAK apical abundance was observed in NRBP1 knockout mice. Representative images of sections co-stained with pSPAK and WNK4 antibodies **(A)** and pSPAK and NCC antibodies **(B)** are presented. **(C)** Representative images of kidney sections from wildtype and NRBP1 knockout mice co-stained with antibodies against SPAK and NCC. **(D)** Results of quantitation of apical pSPAK and SPAK signal. Each dot represents a different animal. Approximately 15 tubules per mice were quantified. **(E)** Results of density analysis of WNK4-positive condensates and pSPAK-positive condensates. Each dot represents a different animal. Approximately 15 tubules per mice were quantified. **(F, G)** Size of WNK-positive (F) and pSPAK-positive (G) condensates was analyzed in male and female mice wildtype and knockout mice. Larger condensates were observed in knockout mice. However, no difference in size was observed between sexes. Each dot represents a different condensate. Results of quantitation are represented in box plots showing median and interquartile range. Student t tests were performed to identify statistically significant differences between genotypes in D and E. For analysis of data presented in F and G, Wilcoxon tests were performed. Scheirer-Ray-Hare tests followed by pairwise Wilcoxon post-hoc tests were performed to evaluate interaction between genotype and sex. $*p<0.05$, $**p<0.01$, $***p<0.001$, $****p<0.0001$

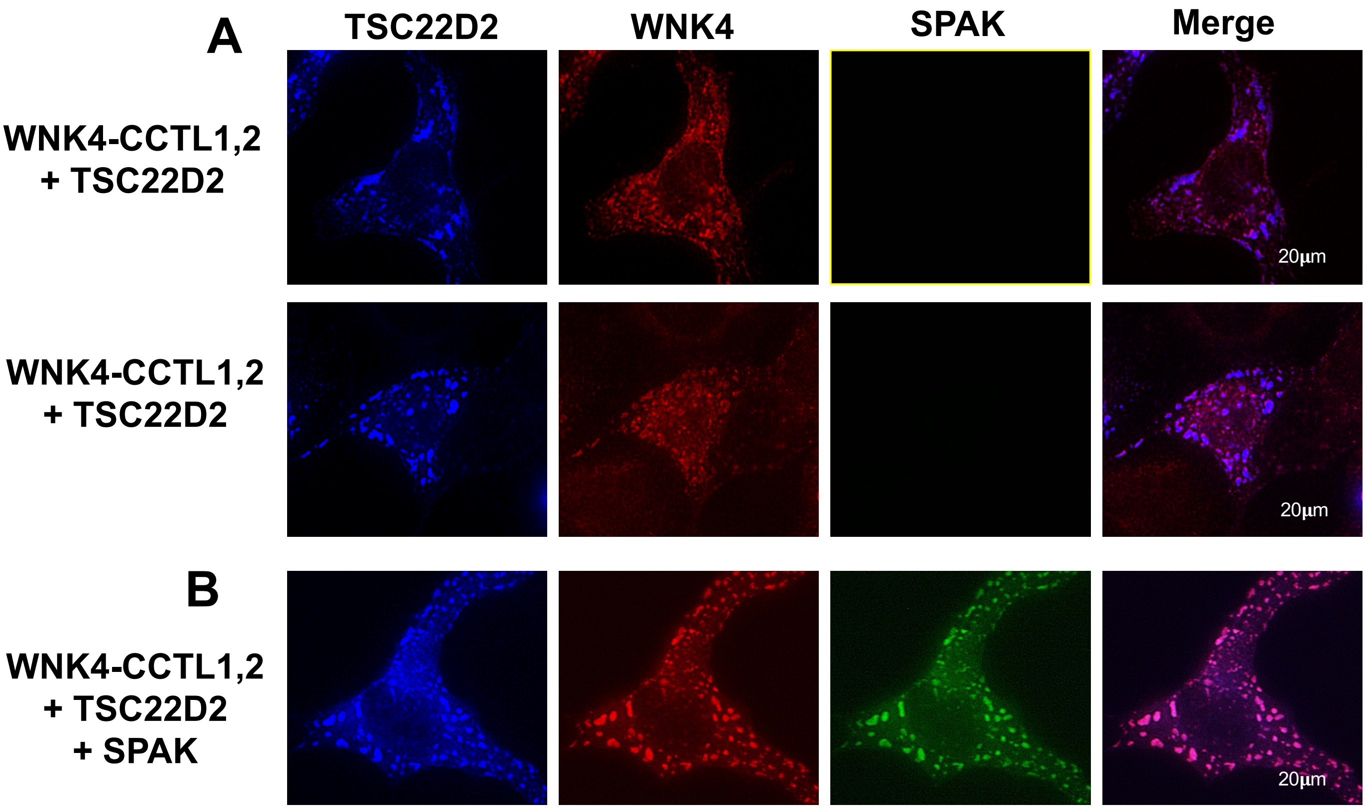

Fig. S7. WNK4-CCTL1,2 mutant does not co-localize with TSC22D2 in cytoplasmic condensates in the absence of SPAK. COS7 cells were transiently transfected with the WNK4-CCTL1,2 mutant. TSC22D2-BFP was co-transfected in the absence **(A)** or presence **(B)** of SPAK-GFP. Panel A shows TSC22D2- condensates surrounded by WNK4 condensates, with no signal overlapping. Panel B shows that in the presence of SPAK, TSC22D2 and the WNK4-CCTL1,2 mutant colocalize in condensates.

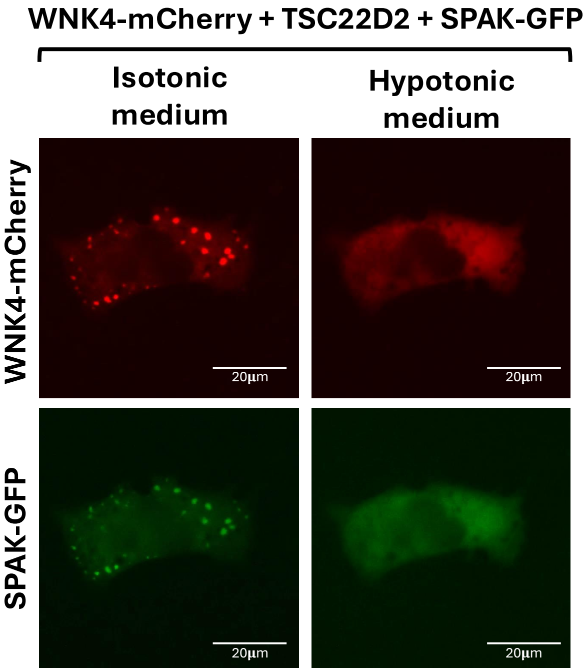

Fig. S8. Condensates formed in response to WNK4 and TSC22D2 overexpression can be dissolved in response to hypotonic stress. COS7 cells were transfected with WNK4-mCherry, TSC22D2, and SPAK-GFP. Forty-eight hours after transfection cells water was added to the media to expose the cells to hypotonic stress (200 mOsm). A time-lapse movie was generated (Video S1). Representative images of the cells before and after 6 minutes of hypotonic stimulation are presented.

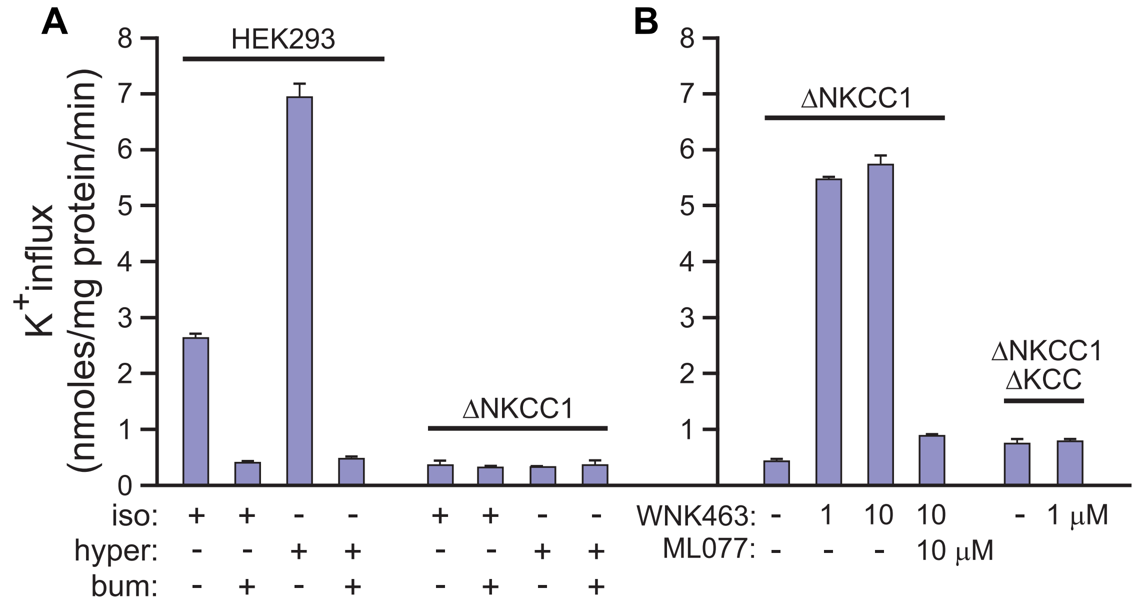

Fig. S9. Validation of *SLC12* knockout cells. (A) ^{83}Rb uptake experiments were performed in wild type HEK293 cells and cells in which the *SLC12A2* gene was targeted by CRISPR-CAS9 (ΔNKCC1 cells). Experiments were performed under isosmotic and hyperosmotic conditions and in the absence or presence of the NKCC inhibitor bumetanide. K^+ influx was calculated and expressed in nanomole K+ per mg protein per min. **(B)** ^{83}Rb uptake experiments were performed in ΔNKCC1 cells previously validated in (A) and in ΔNKCC ΔKCC cells that were generated by simultaneously targeting all KCC-encoding genes in the ΔNKCC1 cells. KCC activity was stimulated by preincubation with the WNK inhibitor WNK463. Uptake experiments were performed in the absence or presence of the KCC inhibitor ML077.

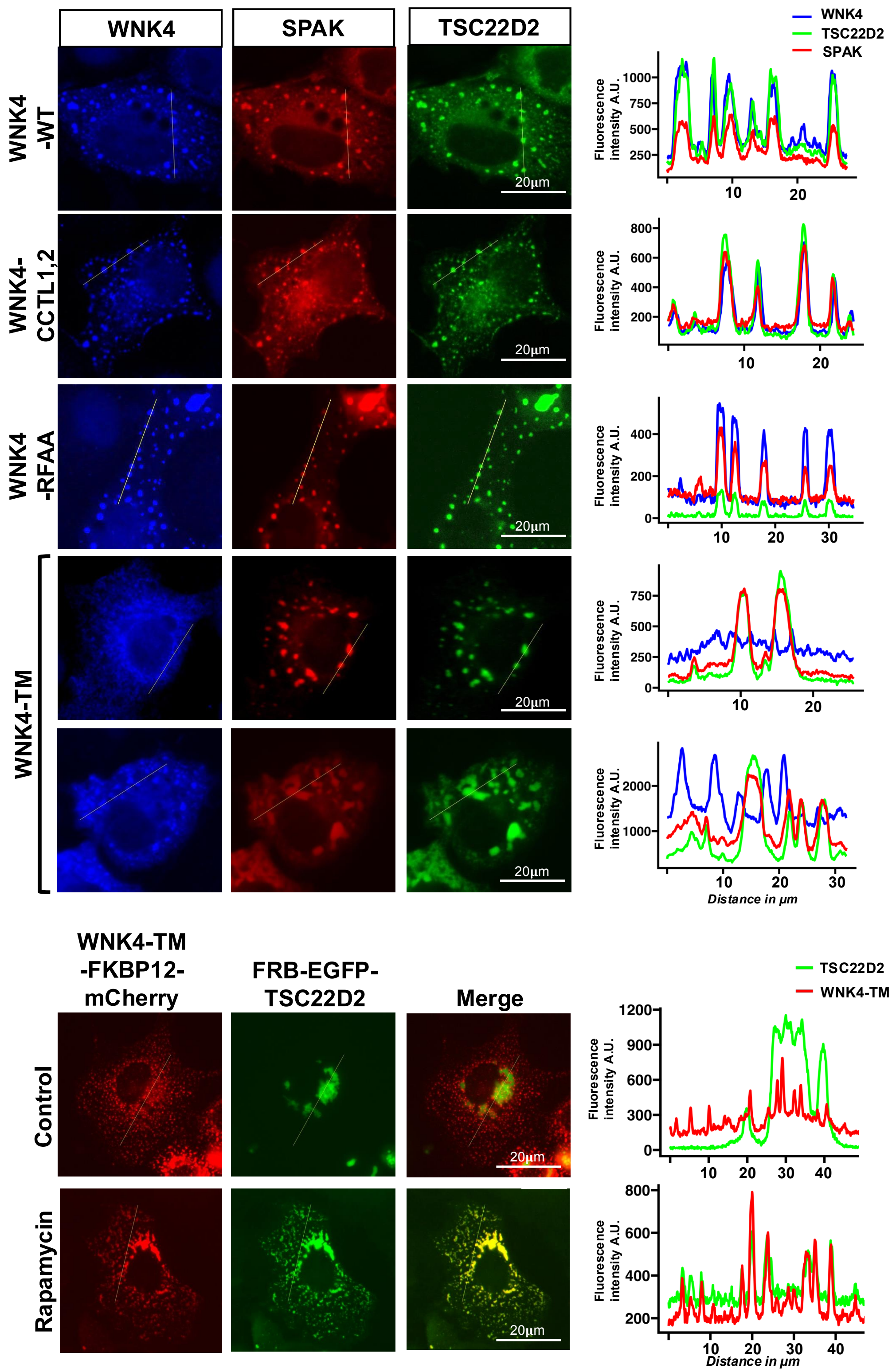

Fig. S10. Colocalization analysis of WNK4 mutants with TSC22D2 and SPAK. Fluorescence intensity within defined linear regions (white lines) of micrographs shown in figure 8 was measured. Graphs to the right show intensity values measured at specific locations within this region. Similar patterns of fluorescence intensity peaks indicate colocalization.

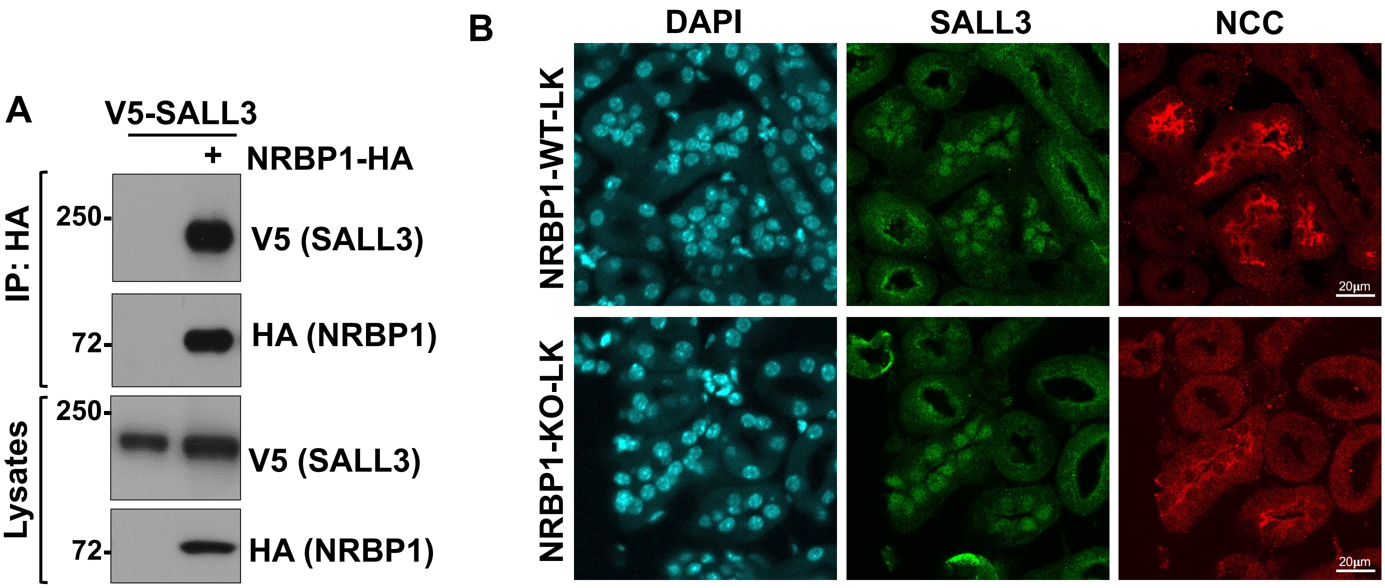

Figure S11. NRBP1 binds SALL3 in vitro. (A) HEK293 cells were transfected with V5-tagged SALL3 and HA-tagged NRBP1. HA-IP was performed to assess SALL3 co-immunoprecipitation. At least three independent experiments were performed with similar results. **(B)** In kidney sections stained with fluorescent probe-tagged antibodies a nuclear signal for SALL3 was only observed in DCT cells as previously reported (1). SALL3 nuclear localization in NRBP1 knockout mice was similar to that observed in control mice.

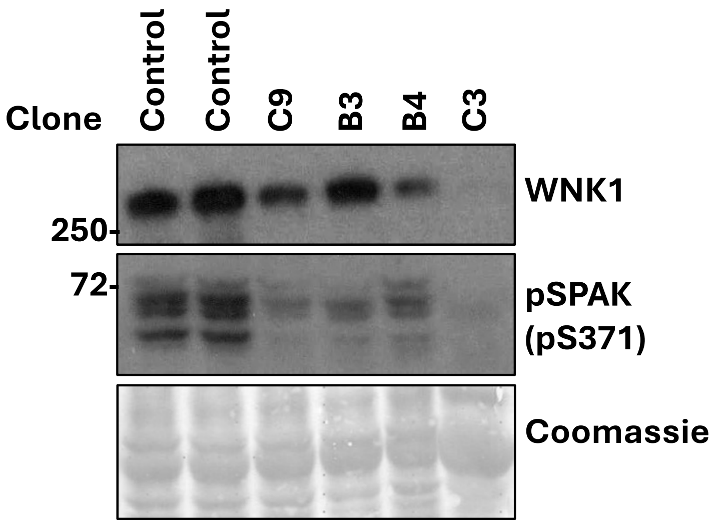

Figure S12. Generation and validation of an L-WNK1-knockout HEK293 cell line. An L-WNK1-knockout HEK293 cell line was generated with CRISPR-Cas9. Control lanes represent parental HEK293 cells. Individual clones, named after the well where they were originally grown, were assessed by Western blot. The C3 clone was identified as the L-WNK1 knockout, displaying undetectable WNK1 protein levels and significantly reduced pSPAK levels.

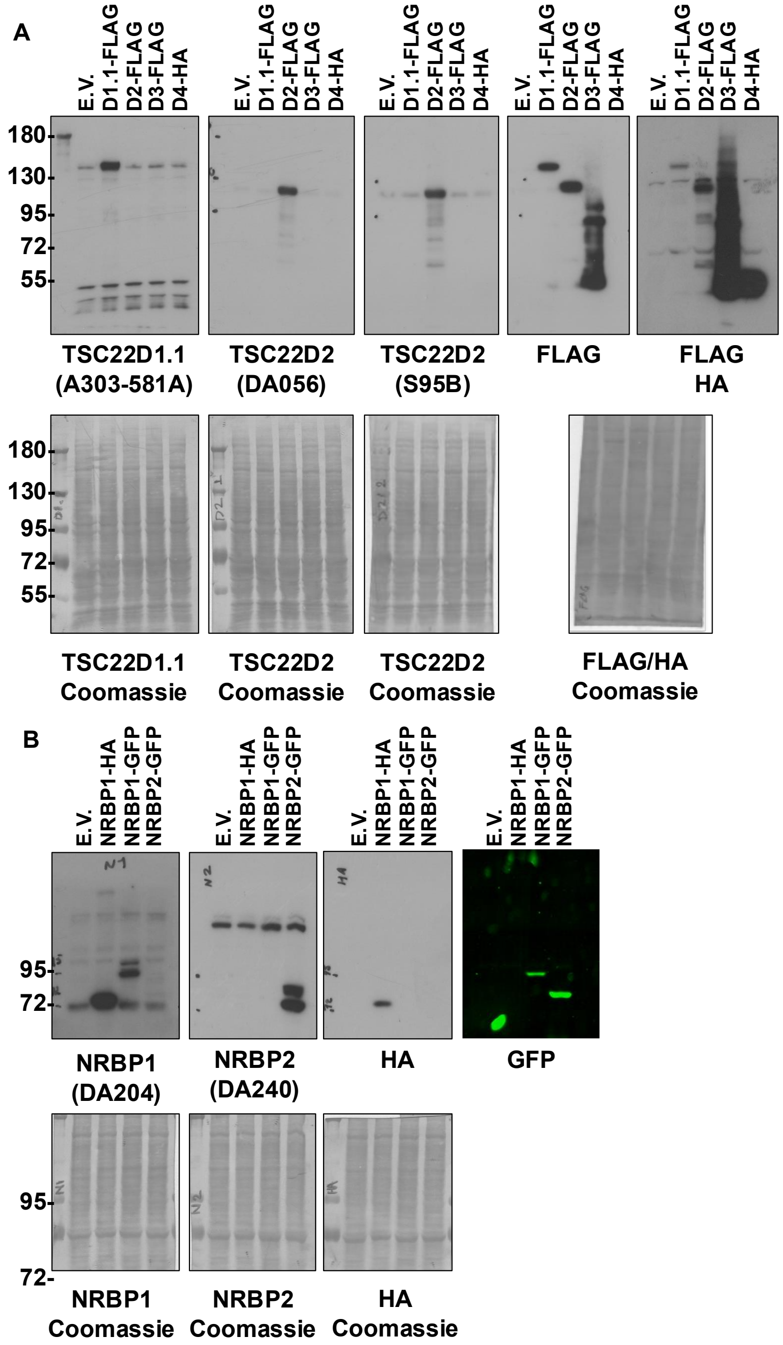

Figure S13. Validation of the TSC22D and NRBP antibodies. HEK293 cells were transfected with the indicated constructs and protein extracts were used to perform immunoblots with the indicated TSC22Ds antibodies **(A)** or NRBP antibodies **(B)**. Results show that the antibodies against each specific TSC22D do not recognize other TSC22D proteins and that the NRBP1 and NRBP2 antibodies do not cross react. Immunofluorescence data presented in Fig. 5 also supports the specificity of the NRBP1 antibody.

Table S1. Plasma electrolytes of DCT-specific NRBP1 knockout mice and their wild type littermates when placed on a low K^+ diet.

	Sex	Mean $NRBP1^{WT}$ (mEq/L)	SD $NRBP1^{WT}$ (mEq/L)	Mean $NRBP1^{KO}$ (mEq/L)	SD $NRBP1^{KO}$ (mEq/L)	n $NRBP1^{WT}$	n $NRBP1^{KO}$
Na^+	Female	150.49	4.06	149.69	4.51	14	14
	Male	148.88	4.75	149.51	5.94	13	7
K^+	Female	2.68	0.6	2.37	0.47	14	14
	Male	2.69	0.6	2.17	0.5	13	7
Cl^-	Female	115.26	3.4	114.99	3.9	14	14
	Male	114.64	3.99	114.84	5.1	13	7

Table S2. Plasmid constructs used

Plasmids	Name	Accession	Mutations	Tag	Species	Origin, ID	Ref.
**pcDNA5D FRT/TO HA NRBP1**	HA-NRBP1	NP_001308286		HA	Human	MRC-PPU-Dundee, DU68362	(*12*)
**pCMV5D HA TSC22D2**	HA TSC22D2	NP_055594		HA	Human	MRC-PPU-Dundee, DU68295	(*12*)
**pcDNA5D FRT/TO FLAG TSC22D2**	FLAG TSC22D2	NP_055594		FLAG	Human	MRC-PPU-Dundee, DU80387	(*12*)
**pCMV5D FLAG TSC22D1**	FLAG TSC22D1	NP_904358		FLAG	Human	MRC-PPU-Dundee, DU68673	(*12*)
**pcDNA5D FRT/TO FLAG TSC22D4**	FLAG TSC22D4	NP_001289972		FLAG	Human	MRC-PPU-Dundee, DU77694	(*12*)
**pCMV5D FLAG TSC22D3**	FLAG TSC22D3	NP_001305397		FLAG	Human	MRC-PPU-Dundee, DU72786	This study
**GFP-HA-SPAK-pEGFPC1**	GFP-HA-SPAK	AF099989.1		GFP HA	Human	MRC-PPU-Dundee, DU6188	(*12*)
**pcDNA 3.1 (-) WNK4 FLAG**	WNK4 FLAG	NP_115763		FLAG	Human	MCB Lab	This study
**pcDNA 3.1 (-) WNK4 HA**	WNK4 HA	AAO21955		HA	Mouse	Lifton Lab	(*51*)
**pcDNA 3.1 (-) WNK4-mCherry -HA**	WNK4-mCherry- HA	AAO21955		HA	Mouse	MCB Lab	This study
**pcDNA 3.1 (-) WNK4 CCTL1 FLAG**	WNK4 CCTL1 FLAG		(F476A, F478A)	FLAG	Human	MCB Lab	This study
**pcDNA 3.1 (-) WNK4 CCTL2 FLAG**	WNK4 CCTL2 FLAG		(V701A, V703A)	FLAG	Human	MCB Lab	This study
**pcDNA 3.1 (-) WNK4 CCTL1,2 FLAG**	WNK4 CCTL1 CCTL2 FLAG		(F476A, F478A) , (V701A, V703A)	FLAG	Human	MCB Lab	This study
**pcDNA 3.1 (-) WNK4 RFAA FLAG**	WNK4 RFAA FLAG		(R1016A, F1017A)	FLAG	Human	MCB Lab	This study
**pcDNA 3.1 (-) WNK4 CCTL1,2 RFAA (WNK4-TM) FLAG**	WNK4 CCTL1 CCTL2 RFAA FLAG		(F476A, F478A) , (V701A, V703A), (R1016A, F1017A)	FLAG	Human	MCB Lab	This study
**mCherry-HA-SPAK**	mCherry-HA-SPAK			HA		MCB Lab	This study
**pcDNA5D FRT/TO GFP TSC22D2**	EGFP TSC22D2	NP_055594				MRC-PPU-Dundee, DU10054	This study
**pcDNA5D FRT/TO BFP TSC22D2**	BFP TSC22D2	NP_055594				MCB Lab	This study
**pCMV5D EGFP V5 TSC22D3**	EGFP V5 TSC22D3	NP_001305397		GFP V5	Human	MCB Lab	This study
**pcDNA 3.1 (-) WNK4-TM-mCherry-FKBP12 FLAG**	WNK4-TM-FKBP12		(F476A, F478A) , (V701A, V703A), (R1016A, F1017A)	FLAG	Human	MCB Lab	This study
**pcDNA5D FRT/TO GFP-FRB-TSC22D2**	TSC22D2-FRB	NP_055594			Human	MCB Lab	This study
**pSpCas9(BB)-2A-GFP (PX458)**	Cas9 2A-EGFP			3x FLAG	S. pyogenes	Addgene #48138 (F. Zhang)	(*44*)
**pcDNA5D FRT/TO GFP TSC22D2-CRY2**	EGFP-TSC22D2--CRY2	NP_055594			Human	MCB Lab	This study

Table S3. Antibodies used

Antibody	Dilution factor	Source	ID	Validation Ref.
**Rabbit Anti-TSC22D1.1**	1:1000 (WB) 1:200 (IF)	Bethyl	A303-581A	This paper (Fig S13)
**Sheep Anti-TSC22D2**	3μg/ml (WB) 1:200 (IF)	MRC-PPU-Dundee	DA056	This paper (Fig S13) and (*12*)
**Sheep Anti-TSC22D2**	3μg/ml (WB) 1:200 (IF)	MRC-PPU-Dundee	S952B	This paper (Fig S13) and (*12*)
**Sheep Anti-NRBP1**	3μg/ml (WB) 1:200 (IF)	MRC-PPU-Dundee	DA204	This paper (Fig 5, S13) and (*12*)
**Rabbit Anti-NRBP1**	1:1000 (WB) 1:200 (IF)	Sigma	HPA029527	This paper (Fig 5 and (*12*)
**Sheep Anti-NRBP2**	3μg/ml (WB) 1:200 (IF)	MRC-PPU-Dundee	DA240	This paper (Fig S13) and (*12*)
**Rabbit Anti-WNK1**	1:2000 (WB) 1:1000 (IF)	Bethyl	A301-515A	(*24*)
**Rabbit Anti-WNK4**	1:5000 (WB & IF)	Ellison Lab		(*47*)
**Rabbit Anti-NCC**	1:5000 (WB & IF)	Ellison Lab		(*52*)
**Sheep Anti-NCC 3P**	3μg/ml	MRC-PPU-Dundee	S908B	(*53*)
**Sheep Anti-WNK1 pS382**	3μg/ml	MRC-PPU-Dundee	S099B	(*30*)
**Sheep Anti-pSPAK S373/ pOSR1 S325**	2 μg/ml (WB) 1:100 (IF)	MRC-PPU-Dundee	S670B	(*25*)
**FLAG**	1:5000	Sigma	A8592	
**HA**<br>**V5**	1:2000 (WB)<br>1:5000 (WB)	Sigma<br>Invitrogen	H6533<br>460708	

Video S1. Condensates formed in response to WNK4 and TSC22D2 overexpression can be dissolved in response to hypotonic stress. Live cell imaging was performed in COS7 cells transfected with WNK4-mCherry, TSC22D2, and SPAK-GFP. Cells were observed before, during (sec 10), and after addition of water to the media to induce hypotonic stress.

Video S2. COS7 cells overexpressing WNK4 and TSC22D2 can form condensates in response to hypertonic stress. Live cell imaging was performed in COS7 cells transfected with WNK4-mCherry and SPAK-GFP. Cells in which no condensates were observed at baseline were identified. The formation of condensates was followed after addition of sorbitol (final concentration 250 mM, sec 5).

Data S1. Densitometric analysis of immunoblots from renal tissue of WNK4 wild-type and WNK4 knockout mice, and KLHL3 wild-type or KLHL3-R528H mutant mice. (Presented in Figure 3).

Data S2. Densitometric analysis of immunoblots from HEK293 cells transfected with SPAK, WNK4, NRBP1, and TSC22D2. (Presented in Figure 4).

Data S3. Densitometric analysis of immunoblots from renal tissue of NRBP1 wild-type and NRBP1 knockout mice. (Presented in Figure 5).

Data S4. Densitometric analysis of immunoblots from HEK293 cells transfected with the WNK4-CCTL1 and WNK4-CCTL2 mutants. (Presented in Figure 6).

Data S5. Densitometric analysis of immunoblots from HEK293 cells co-expressing WNK4-CCTL1/2-RFAA fused to FKBP12 and TSC22D2 fused to FRB. (Presented in Figure 8).

Data S6. Densitometric analysis of immunoblots from HEK293 cells expressing TSC22D3 and pSPAK, and co-expressing TSC22D2-CRY2 with TSC22D3. (Presented in Figure 9).

Data S7. Densitometric analysis of pSPAK levels from HEK293 cells transfected with TSC22D1, TSC22D4, and TSC22D3. (Presented in Supplementary Figure S2).

Data S8. Densitometric analysis of pSPAK levels from HEK293 cells transfected with NRBP2. (Presented in Supplementary Figure S3).

Data S9. Densitometric analysis of pSPAK levels in HEK293 cells expressing NRBP1: dose-response curve. (Presented in Supplementary Figure S4).

Data S10. Quantification of immunofluorescent staining of renal tissue from NRBP1 wild-type and NRBP1 knockout mice for WNK4, SPAK, and pSPAK. (Presented in Supplementary Figure S6).

REFERENCES AND NOTES

1. A. R. Murillo-de-Ozores, M. Chávez-Canales, P. de los Heros, G. Gamba, M. Castañeda-Bueno, Physiological processes modulated by the chloride-sensitive WNK-SPAK/OSR1 kinase signaling pathway and the cation-coupled chloride cotransporters. *Front. Physiol.* **11**, 585907 (2020).

2. F. H. Wilson, S. Disse-Nicodème, K. A. Choate, K. Ishikawa, C. Nelson-Williams, I. Desitter, M. Gunel, D. V. Milford, G. W. Lipkin, J.-M. Achard, M. P. Feely, B. Dussol, Y. Berland, R. J. Unwin, H. Mayan, D. B. Simon, Z. Farfel, X. Jeunemaitre, R. P. Lifton, Human hypertension caused by mutations in WNK kinases. *Science* **293**, 1107–1112 (2001).

3. S. Shibata, J. Zhang, J. Puthumana, K. L. Stone, R. P. Lifton, Kelch-like 3 and Cullin 3 regulate electrolyte homeostasis via ubiquitination and degradation of WNK4. *Proc. Natl. Acad. Sci. U.S.A.* **110**, 7838–7843 (2013).

4. A. Ohta, F. Schumacher, Y. Mehellou, C. Johnson, A. Knebel, T. J. Macartney, N. T. Wood, D. R. Alessi, T. Kurz, The CUL3-KLHL3 E3 ligase complex mutated in Gordon's hypertension syndrome interacts with and ubiquitylates WNK isoforms: Disease-causing mutations in KLHL3 and WNK4 disrupt interaction. *Biochem. J.* **451**, 111–122 (2013).

5. M. D. Lalioti, J. Zhang, H. M. Volkman, K. T. Kahle, K. E. Hoffmann, H. R. Toka, C. Nelson-Williams, D. H. Ellison, R. Flavell, C. J. Booth, Y. Lu, D. S. Geller, R. P. Lifton, Wnk4 controls blood pressure and potassium homeostasis via regulation of mass and activity of the distal convoluted tubule. *Nat. Genet.* **38**, 1124–1132 (2006).

6. P. R. Grimm, R. Coleman, E. Delpire, P. A. Welling, Constitutively active SPAK causes hyperkalemia by activating NCC and remodeling distal tubules. *J. Am. Soc. Nephrol.* **28**, 2597–2606 (2017).

7. M. Castaneda-Bueno, L. G. Cervantes-Perez, N. Vazquez, N. Uribe, S. Kantesaria, L. Morla, N. A. Bobadilla, A. Doucet, D. R. Alessi, G. Gamba, Activation of the renal Na^{+}:Cl^{-} cotransporter by angiotensin II is a WNK4-dependent process. *Proc. Natl. Acad. Sci U.S.A.*. **109**, 7929–7934 (2012).

8. D. Takahashi, T. Mori, N. Nomura, M. Z. H. Khan, Y. Araki, M. Zeniya, E. Sohara, T. Rai, S. Sasaki, S. Uchida, WNK4 is the major WNK positively regulating NCC in the mouse kidney. *Biosci. Rep.* **34**, 195–206 (2014).

9. K. Susa, E. Sohara, D. Takahashi, T. Okado, T. Rai, S. Uchida, WNK4 is indispensable for the pathogenesis of pseudohypoaldosteronism type II caused by mutant KLHL3. *Biochem. Biophys. Res. Commun.* **491**, 727–732 (2017).

10. E. Vidal-Petiot, L. Cheval, J. Faugeroux, T. Malard, A. Doucet, X. Jeunemaitre, J. Hadchouel, A new methodology for quantification of alternatively spliced exons reveals a highly tissue-specific expression pattern of WNK1 isoforms. *PLOS ONE* **7**, e37751 (2012).

11. Y.-X. Xiao, S. Y. Lee, M. Aguilera-Uribe, R. Samson, A. Au, Y. Khanna, Z. Liu, R. Cheng, K. Aulakh, J. Wei, A. G. Farias, T. Reilly, S. Birkadze, A. Habsid, K. R. Brown, K. Chan, P. Mero, J. Q. Huang, M. Billmann, M. Rahman, C. Myers, B. J. Andrews, J.-Y. Youn, C. M. Yip, D. Rotin, W. B. Derry, J. D. Forman-Kay, A. M. Moses, I. Pritišanac, A.-C. Gingras, J. Moffat, The TSC22D, WNK, and NRBP gene families exhibit functional buffering and evolved with Metazoa for cell volume regulation. *Cell Rep.* **43**, 114417 (2024).

12. R. Amnekar, T. Dite, P. Lis, S. Bell, F. Brown, C. Johnson, S. Wilkinson, S. Raggett, M. Dorward, M. Wightman, T. Macartney, R. F. Soares, F. Lamoliatte, D. R. Alessi, NRBP1 pseudokinase binds to and activates the WNK pathway in response to osmotic stress. bioRxiv 628181 [Preprint] (2024). https://doi.org/10.1101/2024.12.12.628181.

13. S. Gluderer, E. Brunner, M. Germann, V. Jovaisaite, C. Li, C. A. Rentsch, E. Hafen, H. Stocker, Madm (Mlf1 adapter molecule) cooperates with Bunched A to promote growth in *Drosophila*. *J. Biol.* **9**, 9 (2010).

14. G. Manning, D. B. Whyte, R. Martinez, T. Hunter, S. Sudarsanam, The protein kinase complement of the human genome. *Science* **298**, 1912–1934 (2002).

15. C. A. Taylor, J.-U. Jung, S. G. Kankanamalage, J. Li, M. Grzemska, A. B. Jaykumar, S. Earnest, S. Stippec, P. Saha, E. Sauceda, M. H. Cobb, Predictive and experimental motif

interaction analysis identifies functions of the WNK-OSR1/SPAK pathway. bioRxiv 600905 [Preprint] (2024). https://doi.org/10.1101/2024.06.26.600905.

16. A. R. Murillo-de-Ozores, A. Rodríguez-Gama, H. Carbajal-Contreras, G. Gamba, M. Castañeda-Bueno, WNK4 kinase: From structure to physiology. *Am. J. Physiol. Physiol.* **320**, F378–F403 (2021).

17. K. B. Gagnon, E. Delpire, Molecular physiology of SPAK and OSR1: Two Ste20-related protein kinases regulating ion transport. *Physiol. Rev.* **92**, 1577–1617 (2012).

18. F. Villa, J. Goebel, F. H. Rafiqi, M. Deak, J. Thastrup, D. R. Alessi, D. M. F. van Aalten, Structural insights into the recognition of substrates and activators by the OSR1 kinase. *EMBO Rep.* **8**, 839–845 (2007).

19. R. Lim, L. N. Winteringham, J. H. Williams, R. K. McCulloch, E. Ingley, J. Y. H. Tiao, J. P. Lalonde, S. Tsai, P. A. Tilbrook, Y. Sun, X. Wu, S. W. Morris, S. Peter Klinken, MADM, a novel adaptor protein that mediates phosphorylation of the 14-3-3 binding site of myeloid leukemia factor 1. *J. Biol. Chem.* **277**, 40997–41008 (2002).

20. N. Mahrour, W. B. Redwine, L. Florens, S. K. Swanson, S. Martin-Brown, W. D. Bradford, K. Staehling-Hampton, M. P. Washburn, R. C. Conaway, J. W. Conaway, Characterization of Cullin-box sequences that direct recruitment of Cul2-Rbx1 and Cul5-Rbx2 modules to Elongin BC-based ubiquitin ligases. *J. Biol. Chem.* **283**, 8005–8013 (2008).

21. T. Yasukawa, A. Tsutsui, C. Tomomori-Sato, S. Sato, A. Saraf, M. P. Washburn, L. Florens, T. Terada, K. Shimizu, R. C. Conaway, J. W. Conaway, T. Aso, NRBP1-containing CRL2/CRL4A regulates amyloid β production by targeting BRI2 and BRI3 for degradation. *Cell Rep.* **30**, 3478–3491.e6 (2020).

22. C. R. Boyd-Shiwarski, D. J. Shiwarski, S. E. Griffiths, R. T. Beacham, L. Norrell, D. E. Morrison, J. Wang, J. Mann, W. Tennant, E. N. Anderson, J. Franks, M. Calderon, K. A. Connolly, M. U. Cheema, C. J. Weaver, L. J. Nkashama, C. C. Weckerly, K. E. Querry, U. B. Pandey, C. J. Donnelly, D. Sun, A. R. Rodan, A. R. Subramanya, WNK kinases sense

molecular crowding and rescue cell volume via phase separation. *Cell* **185**, 4488–4506.e20 (2022).

23. C. R. Boyd-Shiwarski, D. J. Shiwarski, A. Roy, H. N. Namboodiri, L. J. Nkashama, J. Xie, K. L. McClain, A. Marciszyn, T. R. Kleyman, R. J. Tan, D. B. Stolz, M. A. Puthenveedu, C. L. Huang, A. R. Subramanya, Potassium-regulated distal tubule WNK bodies are kidney-specific WNK1 dependent. *Mol. Biol. Cell* **29**, 499–509 (2018).

24. M. Ostrosky-Frid, M. Chávez-Canales, J. Zhang, O. Andrukhova, E. R. Argaiz, F. Lerdo-de-Tejada, A. Murillo-de-Ozores, A. Sanchez-Navarro, L. Rojas-Vega, N. A. Bobadilla, N. Vazquez, M. Castañeda-Bueno, D. R. Alessi, G. Gamba, Role of KLHL3 and dietary K^+ in regulating KS-WNK1 expression. *Am. J. Physiol. Physiol.* **320**, F734–F747 (2021).

25. M. N. Thomson, C. A. Cuevas, T. M. Bewarder, C. Dittmayer, L. N. Miller, J. Si, R. J. Cornelius, X.-T. Su, C.-L. Yang, J. A. McCormick, J. Hadchouel, D. H. Ellison, S. Bachmann, K. Mutig, WNK bodies cluster WNK4 and SPAK/OSR1 to promote NCC activation in hypokalemia. *Am. J. Physiol. Physiol.* **318**, F216–F228 (2020).

26. L. Chen, C. Chou, M. A. Knepper, A comprehensive map of mRNAs and their isoforms across all 14 renal tubule segments of mouse. *J. Am. Soc. Nephrol.* **32**, 897–912 (2021).

27. K. Limbutara, C.-L. Chou, M. A. Knepper, Quantitative proteomics of all 14 renal tubule segments in rat. *J. Am. Soc. Nephrol.* **31**, 1255–1266 (2020).

28. P. Rashmi, G. L. Colussi, M. Ng, X. Wu, A. Kidwai, D. Pearce, Glucocorticoid-induced leucine zipper protein regulates sodium and potassium balance in the distal nephron. *Kidney Int.* **91**, 1159–1177 (2017).

29. A. R. Murillo-de-Ozores, H. Carbajal-Contreras, G. R. Magaña-Ávila, R. Valdés, L. I. Grajeda-Medina, N. Vázquez, T. Zariñán, A. López-Saavedra, A. Sharma, D.-H. Lin, W.-H. Wang, E. Delpire, D. H. Ellison, G. Gamba, M. Castañeda-Bueno, Multiple molecular mechanisms are involved in the activation of the kidney sodium-chloride cotransporter by hypokalemia. *Kidney Int.* **102**, 1030–1041 (2022).

30. A. Zagórska, E. Pozo-Guisado, J. Boudeau, A. C. Vitari, F. H. Rafiqi, J. Thastrup, M. Deak, D. G. Campbell, N. A. Morrice, A. R. Prescott, D. R. Alessi, Regulation of activity and localization of the WNK1 protein kinase by hyperosmotic stress. *J. Cell Biol.* **176**, 89–100 (2007).

31. R. J. Cornelius, A. Sharma, X. T. Su, J. J. Guo, J. A. McMahon, D. H. Ellison, A. P. McMahon, J. A. McCormick, A novel distal convoluted tubule-specific Cre-recombinase driven by the NaCl cotransporter gene. *Am. J. Physiol. Ren. Physiol.* **319**, F423–F435 (2020).

32. F. H. Rafiqi, A. M. Zuber, M. Glouer, C. Richardson, S. Fleming, S. Jouanouić, A. Jouanouić, M. O. S. Kevin, D. R. Alessi, Role of the WNK-activated SPAK kinase in regulating blood pressure. *EMBO Mol. Med.* **2**, 63–75 (2010).

33. A. C. Vitari, J. Thastrup, F. H. Rafiqi, M. Deak, N. A. Morrice, H. K. R. Karlsson, D. R. Alessi, Functional interactions of the SPAK/OSR1 kinases with their upstream activator WNK1 and downstream substrate NKCC1. *Biochem. J.* **397**, 223–231 (2006).

34. D. Pacheco-Alvarez, N. Vázquez, M. Castañeda-Bueno, P. De-Los-Heros, C. Cortes-González, E. Moreno, P. Meade, N. A. Bobadilla, G. Gamba, WNK3-SPAK interaction is required for the modulation of NCC and other members of the SLC12 family. *Cell. Physiol. Biochem.* **29**, 291–302 (2012).

35. C. A. Taylor, M. H. Cobb, CCT and CCT-like modular protein interaction domains in WNK signaling. *Mol. Pharmacol.* **101**, 201–212 (2022).

36. H. Park, N. Y. Kim, S. Lee, N. Kim, J. Kim, W. Do Heo, Optogenetic protein clustering through fluorescent protein tagging and extension of CRY2. *Nat. Commun.* **8**, 30 (2017).

37. M. Z. Ferdaus, R. B. Koumangoye, P. A. Welling, E. Delpire, Kinase scaffold Cab39 is necessary for phospho-activation of the thiazide-sensitive NCC. *Hypertension* **81**, 801–810 (2024).

38. C. H. Wilson, C. Crombie, L. Van Der Weyden, G. Poulogiannis, A. G. Rust, M. Pardo, T. Gracia, L. Yu, J. Choudhary, G. B. Poulin, R. E. McIntyre, D. J. Winton, H. N. March, M. J.

Arends, A. G. Fraser, D. J. Adams, Nuclear receptor binding protein 1 regulates intestinal progenitor cell homeostasis and tumour formation. *EMBO J.* **31**, 2486–2497 (2012).

39. J. Hwang, M. A. Haque, H. Suzuki, P. ten Dijke, M. Kato, THG-1 suppresses SALL4 degradation to induce stemness genes and tumorsphere formation through antagonizing NRBP1 in squamous cell carcinoma cells. *Biochem. Biophys. Res. Commun.* **523**, 307–314 (2020).

40. H. J. Jung, B. Kim, M. Z. Ferdaus, L. Al-Qusairi, R. Grimm, E. J. Delpire, P. A. Welling, Spalt-like transcription factor 3 (Sall3) is essential for maintaining distal convoluted tubule differentiation. *J. Am. Soc. Nephrol.* **35**, 10.1681/ASN.202408bh48v6 (2024).

41. Y. Liao, Z. Yang, J. Huang, H. Chen, J. Xiang, S. Li, C. Chen, X. He, F. Lin, Z. Yang, J. Wang, Nuclear receptor binding protein 1 correlates with better prognosis and induces caspase-dependent intrinsic apoptosis through the JNK signalling pathway in colorectal cancer. *Cell Death Dis.* **9**, 436 (2018).

42. S. Gluderer, S. Oldham, F. Rintelen, A. Sulzer, C. Schütt, X. Wu, L. A. Raftery, E. Hafen, H. Stocker, Bunched, the *Drosophila* homolog of the mammalian tumor suppressor TSC-22, promotes cellular growth. *BMC Dev. Biol.* **8**, 10 (2008).

43. H. Jin, C. Zhang, M. Zwahlen, K. von Feilitzen, M. Karlsson, M. Shi, M. Yuan, X. Song, X. Li, H. Yang, H. Turkez, L. Fagerberg, M. Uhlén, A. Mardinoglu, Systematic transcriptional analysis of human cell lines for gene expression landscape and tumor representation. *Nat. Commun.* **14**, 5417 (2023).

44. F. A. Ran, P. D. Hsu, J. Wright, V. Agarwala, D. A. Scott, F. Zhang, Genome engineering using the CRISPR-Cas9 system. *Nat. Protoc.* **8**, 2281–2308 (2013).

45. C. Li, A. Wen, B. Shen, J. Lu, Y. Huang, Y. Chang, FastCloning: A highly simplified, purification-free, sequence- and ligation-independent PCR cloning method. *BMC Biotechnol.* **11**, 92 (2011).

46. J. O. Thastrup, F. H. Rafiqi, A. C. Vitari, E. Pozo-Guisado, M. Deak, Y. Mehellou, D. R. Alessi, SPAK/OSR1 regulate NKCC1 and WNK activity: Analysis of WNK isoform interactions and activation by T-loop trans-autophosphorylation. *Biochem. J.* **441**, 325–337 (2012).

47. A. R. Murillo-de-Ozores, A. Rodríguez-Gama, S. Bazúa-Valenti, K. Leyva-Ríos, N. Vázquez, D. Pacheco-Álvarez, I. A. De La Rosa-Velázquez, A. Wengi, K. L. Stone, J. Zhang, J. Loffing, R. P. Lifton, C. L. Yang, D. H. Ellison, G. Gamba, M. Castañeda-Bueno, C-terminally truncated, kidney-specific variants of the WNK4 kinase lack several sites that regulate its activity. *J. Biol. Chem.* **293**, 12209–12221 (2018).

48. L. R. Teixeira, R. Akella, J. M. Humphreys, H. He, E. J. Goldsmith, Water and chloride as allosteric inhibitors in WNK kinase osmosensing. *eLife* **12**, RP88224 (2024).

49. K. Piechotta, J. Lu, E. Delpire, Cation chloride cotransporters interact with the stress-related kinases Ste20-related proline-alanine-rich kinase (SPAK) and oxidative stress response 1 (OSR1). *J. Biol. Chem.* **277**, 50812–50819 (2002).

50. K. Piechotta, N. Garbarini, R. England, E. Delpire, Characterization of the interaction of the stress kinase SPAK with the Na^+-K^+-$2Cl^-$ cotransporter in the nervous system: Evidence for a scaffolding role of the kinase. *J. Biol. Chem.* **278**, 52848–52856 (2003).

51. M. Castañeda-Bueno, J. P. Arroyo, J. Zhang, J. Puthumana, O. Yarborough, S. Shibata, L. Rojas-Vega, G. Gamba, J. Rinehart, R. P. Lifton, Phosphorylation by PKC and PKA regulate the kinase activity and downstream signaling of WNK4. *Proc. Natl. Acad. Sci. U.S.A.* **114**, E879–E886 (2017).

52. R. J. Cornelius, J. Si, C. A. Cuevas, J. W. Nelson, B. D. K. Gratreak, R. Pardi, C.-L. Yang, D. H. Ellison, Renal COP9 signalosome deficiency alters CUL3-KLHL3-WNK signaling pathway. *J. Am. Soc. Nephrol.* **29**, 2627–2640 (2018).

53. H. Carbajal-Contreras, A. R. Murillo-De-Ozores, G. Magaña-Avila, A. Marquez-Salinas, L. Bourqui, M. Tellez-Sutterlin, J. P. Bahena-Lopez, E. Cortes-Arroyo, S. G. Behn-Eschenburg, A. Lopez-Saavedra, N. Vazquez, D. H. Ellison, J. Loffing, G. Gamba, M. Castañeda-Bueno,

Arginine vasopressin regulates the renal Na^{+}-Cl^{-} and Na^{+}-K^{+}-Cl^{-} cotransporters through with-no-lysine kinase 4 and inhibitor 1 phosphorylation. *Am. J. Physiol. Renal Physiol.* **326**, F285–F299 (2024).
